# Effect of injection of different doses of isoproterenol on the hearts of mice

**DOI:** 10.1186/s12872-022-02852-x

**Published:** 2022-09-12

**Authors:** Yujing Pan, Jin Gao, Renyun Gu, Wanzhen Song, Haoyang Li, Junpeng Wang, Yihuang Gu, Hao Chen, Hongru Zhang

**Affiliations:** 1 School of Acupuncture and Massage, Nanjing University of Chinese Medicine, Nanjing, 210023 China; 2 School of Integrated Chinese and Western Medicine, Nanjing University of Chinese Medicine, Nanjing, 210023 China; 3 First Clinical Medical College, Nanjing University of Chinese Medicine, Nanjing, 210023 China; 4 College of Pharmacy, Nanjing University of Chinese Medicine, Nanjing, 210023 China

**Keywords:** Heart failure, Animal model, Isoproterenol, Dosage standards, Modeling success criteria

## Abstract

**Background:**

Heart failure (HF) is one of the diseases that seriously threaten human health today and its mechanisms are very complex. Our study aims to confirm the optimal dose ISO-induced chronic heart failure mice model for better study of HF-related mechanisms and treatments in the future.

**Methods:**

C57BL/6 mice were used to establish mice model of chronic heart failure. We injected isoproterenol subcutaneously in a dose gradient of 250 mg/kg, 200 mg/kg, 150 mg/kg, 100 mg/kg and 50 mg/kg. Echocardiography and ELISA were performed to figure out the occurrence of HF. We also supplemented the echocardiographic changes in mice over 30 days.

**Results:**

Except group S and group E, echocardiographic abnormalities were found in other groups, suggesting a decrease in cardiac function. Except group S, myofibrolysis were found in the hearts of mice in other groups. Brain natriuretic peptide was significantly increased in groups B and D, and C-reactive protein was significantly increased in each group.

**Conclusion:**

Our research finally found that the HFrEF mice model created by injection at a dose of 100 mg/kg for 7 days was the most suitable and a relatively stable chronic heart failure model could be obtained by placing it for 21 days.

**Supplementary Information:**

The online version contains supplementary material available at 10.1186/s12872-022-02852-x.

## Introduction

Heart failure(HF) is a group of complex clinical syndromes caused by abnormal changes of cardiac structure and/or function, resulting in dysfunction of ventricular systolic and/or diastolic function [1]. The main manifestations are dyspnea, fatigue, fluid retention (pulmonary congestion, systemic blood stasis and peripheral edema) and so on [2]. Brain natriuretic peptide (BNP) and N-terminal proBNP(NT-proBNP) are widely used as diagnostic biomarkers for HF and cardiac dysfunction in clinical medicine [3]. It was long considered as an incurable disease with little hope of recovery [4]. With the development of hemodynamics, neurohormones and effective treatments, heart failure has been transformed into a chronic disease. Chronic heart failure (CHF) is a kind of clinical cardiovascular disease seriously endangering human health and its morbidity and mortality are increasing year by year. The high incidence, poor prognosis and relapse of CHF lead to more and more hospitalizations, undertreatment and higher economic costs [5–9]. Basic animal experiments are essential for CHF research.

At present, the commonly used animal models for the study of CHF are mainly divided into four categories, mainly drug injection, surgery, hypertension model outcome and genetic technology. Each method has its own advantages and disadvantages: There are a variety of surgical modeling methods, mainly ischemic injury and LV pressure overload [10], which can simulate the mechanism of different etiologies to develop CHF. The representative method of ischemic HF model is coronary artery ligation, which was used to mimic myocardial infarction [11]. Ligation of the left anterior descending artery results in HF developing by 6 weeks after infarction. The representative method of pressure overload model is transverse aortic coarctation (TAC), which can simulate HF caused by hypertension [12]. TAC causes an increase in LV afterload, giving rise to concentric hypertrophy, interstitial fibrosis and increasing LV stiffness, eventually leading to systolic failure [13]. However, both methods have the disadvantages of high cost, high operator requirements, high postoperative infection rate and high mortality rate [14]. Genetic techniques are suitable for exploring the etiology of HF but the price is high. More importantly, it cannot reflect the actual disease process of patients [15]. The two most popular methods to generate whole-body gene deletions and conditional knockouts are Cre/loxP and Flippase/FRT-mediated recombination methods [16]. Hypertension-induced CHF model does not require additional interventions but the modeling time is too long [17, 18].Drug induction mainly includes doxorubicin and isoproterenol, which have the advantages of easy operation, short modeling time and low infection rate [19, 20]. For doxorubicin, its mortality rate is higher and it is not in line with the etiology of most HF patients while for isoproterenol, it is affected by animal batch, drug batch, route of administration, etc. [21]. In addition, mice after doxorubicin injection experience toxic adverse effects in their bone marrow and gastrointestinal systems, making this model less than ideal for the investigation of immunologic impacts on HF [22]. There are many different doses in existing articles and this study aims to address the standardization of isoproterenol-induced CHF animal models [23].

## Methods

### Animal procedures

Male C57BL/6 mice were purchased from Nanjing Qingzilan Biotechnology Co.,Ltd. The mice were randomly divided into 6 groups with 6 mice in each group: Group A(250 mg/kg subcutaneous injection) [24], Group B(200 mg/kg subcutaneous injection) [25], Group C(150 mg/kg subcutaneous injection) [26], Group D(100 mg/kg subcutaneous injection) [27], Group E(50 mg/kg subcutaneous injection) and Group S(normal saline group) [28]. According to the above groups, mice were injected with different doses of ISO (concentration: 100 mg/ml,dissolved in normal saline). Animals were randomized per cage, with all in the same cage receiving the same treatment. Investigators were not blinded to treatment group allocation. Housing and procedure rooms were under specific pathogen-free conditions. The mice had a 12-h day/night cycle, with daytime being from 7 am to 7 pm. All animals in this study received humane care in compliance with the “Guide for the Care and Use of Laboratory Animals” published by the US National Institutes of Health (NIH Publication No. 85–23, revised 1996). All animal experiments were performed at the Key Laboratory of Acupuncture and Medicine, Ministry of Education, Nanjing University of Chinese Medicine and were approved by the Animal Ethics Committee, Laboratory Animal Center, Nanjing University of Chinese Medicine(procedure protocol:202103A039). This study involved no human subject research.

### Echocardiography

Mice were anesthetized with 5% isoflurane with high purity oxygen and maintained at concentration of 1%-2%. The mice were placed supine and tilted 30° to the right and the chest hair was culled. Apply ultrasound coupling agent to the chest and place the ultrasound probe on the left side of the sternum. Record test results including left ventricular ejection fraction (LVEF), fraction shortening (FS) and heart rate (HR).

### Histological measurements

After surgical removal of mice hearts, the hearts were flushed with PBS and fixed with 4% paraformaldehyde. The hearts were paraffin embedded and cross sectioned at 5-μm thickness for haematoxylin and eosin staining (H&E staining). The specimens were photographed by a microscope and the relevant sites were collected and analyzed.

### Enzyme linked immunosorbent assay (ELISA)

Mice serum was collected for the measurement of cytokines secretion using ELISA kits (mlbio) according to the manufacturer’s instructions.

### Data and statistical analysis

The data and statistical analysis comply with the recommendations of the British Journal of Pharmacology on experimental design and analysis in pharmacology. All the animal experiments were designed to generate groups of equal size, using randomization and blinded analysis. Data are expressed as the mean ± SEM and GraphPad Prism software was used for statistical analysis. One-way ANOVA followed by post-hoc test adjustments using Bonferroni correction for comparisons among more than two groups. Post-hoc tests were run only if F achieved *P* < 0.05 and there was no significant variance inhomogeneity. *P* < 0.05 was considered to represent a significant difference between group means.

### Materials

Inhalation anesthesia machine for small animals (Shenzhen Reward Life Technology Co., Ltd.), Doppler Ultrasound (Esaote), Desktop high-speed refrigerated centrifuge (Shanghai Anting Scientific Instrument Factory), Microscope (Nikon), Electronic analytical balance (Shanghai Precision Scientific Instrument Co., Ltd.), high purity oxygen (Nanjing Chuangda Special Gas Co., Ltd.), Isoflurane (Shenzhen Reward Life Technology Co., Ltd.).

## Results

### Primary outcome measures

#### ISO causes echocardiographic changes in mice, mainly decreased ejection fraction and changes in heart rate

**Fig. 1:**
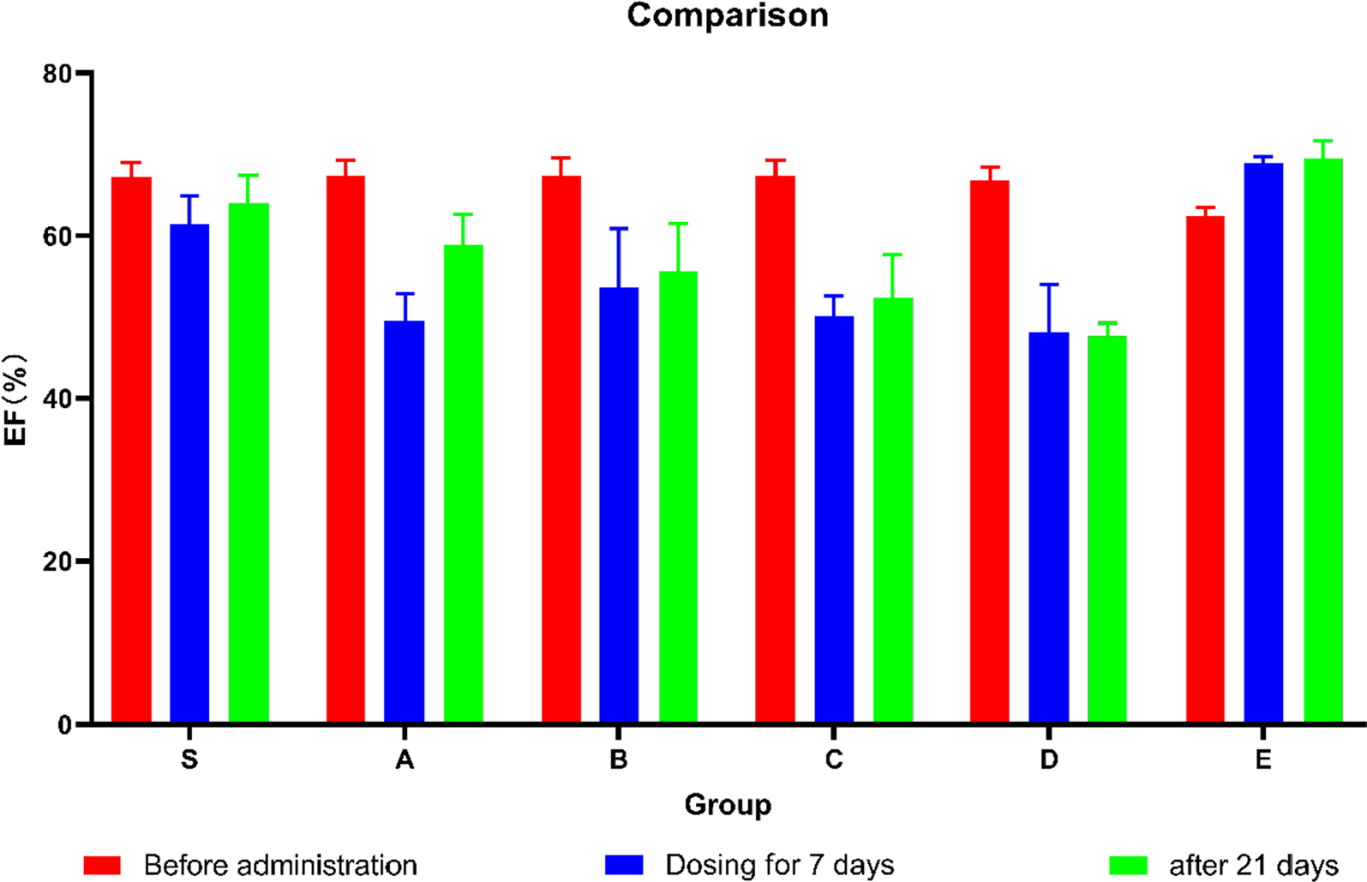
The figure showing the EF before administration (Red), EF of dosing for 7 days (Blue) and EF after 21 days (Green) in groups

**Fig. 2:**
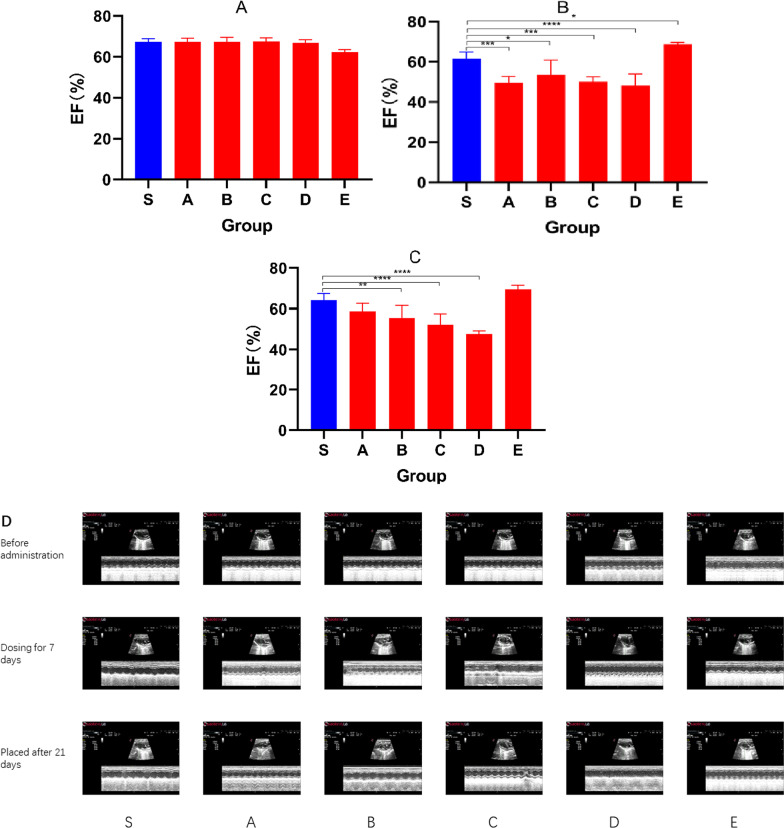
Bar graphs showing the EF before administration (**A**), EF after dosing for 7 days (**B**), EF after 21 days (**C**). (D)Echocardiogram showing FS and EF at 3 times in each group. Data shown are means ± SD; n = 6 in each group. *P < 0.05, significantly different as indicated

The main diagnostic criterion for HFrEF is decreased ejection fraction, which is closely related to cardiac function. Our study found that group A, group B, group C and group D all had decreased ejection fraction. In Fig. 1, the graph shows the changes in EF after different events. Figure 2A shows that there was no statistical difference in the EF of the initially healthy mice (*P* > 0.05). After 7 injections (Fig. 2B), the EF of the E group increased and the others decreased. After 21 days of placement (Fig. 2C), the other model groups also showed fluctuations in EF. Figure 2D is a recorded echocardiogram and the original images have been in Additional file 1.

**Fig. 3:**
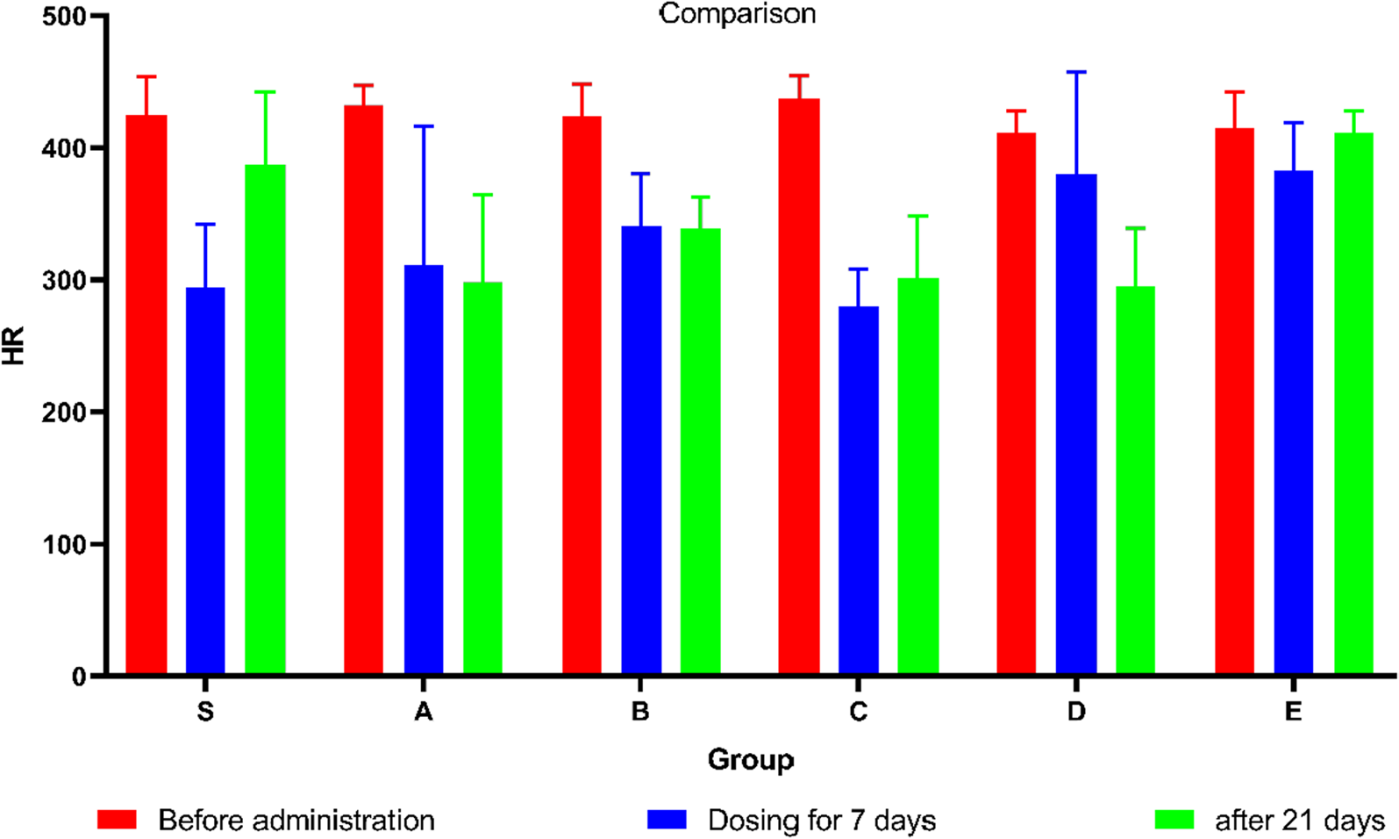
The figure showing the HR before administration (Blue bar graph), HR of dosing for 7 days (Red bar graph) and HR after 21 days (Green bar graph) in groups

**Fig. 4:**
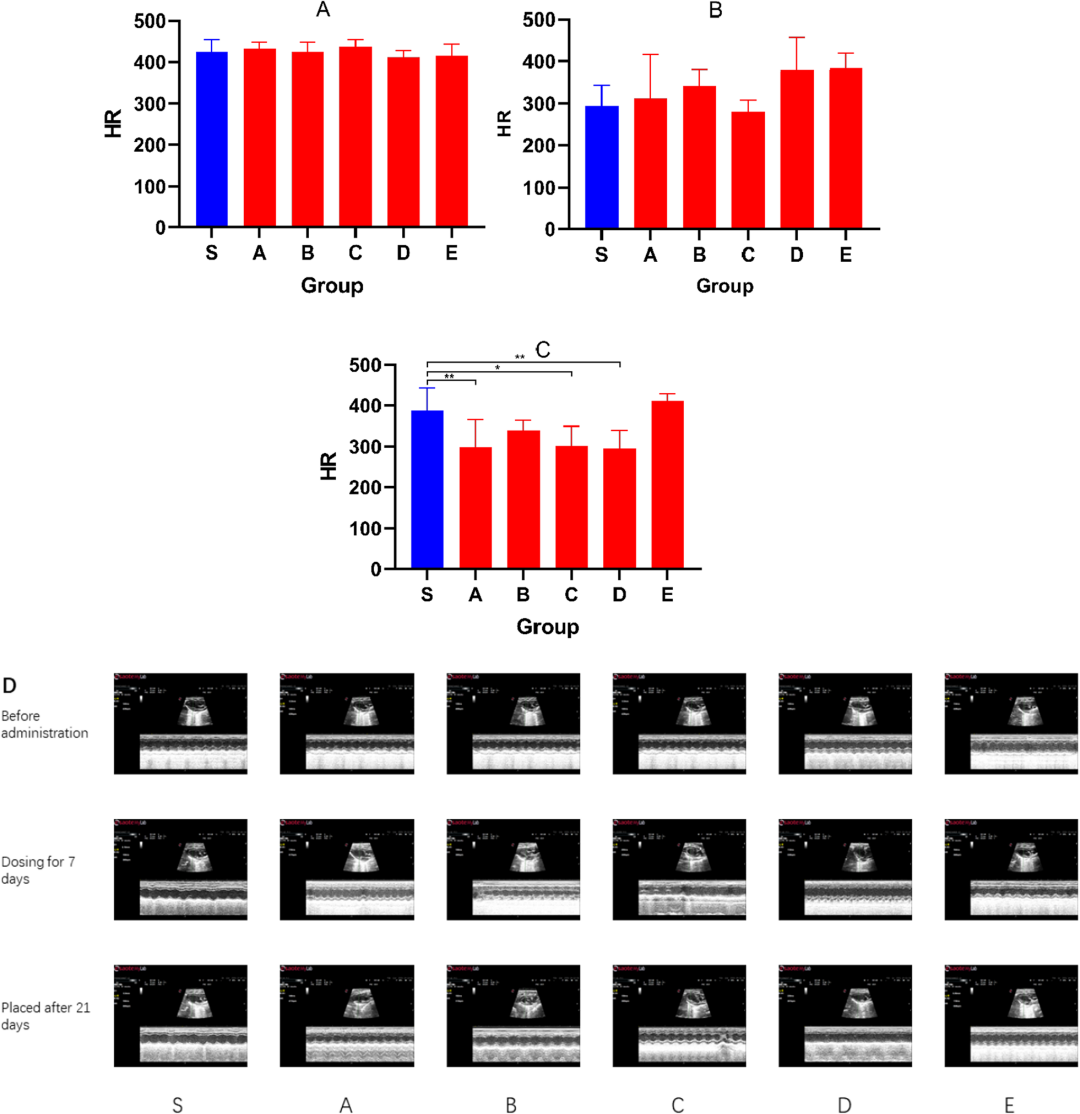
Bar graphs showing the HR before administration (**A**), HR after dosing for 7 days (**B**), HR after 21 days (**C**). (**D**) Echocardiogram showing HR at 3 times in each group. Data shown are means ± SD; n = 6 in each group. *P < 0.05,significantly different as indicated

Important indicators of echocardiography also include HR reflecting changes in cardiac function. Figure 3 shows the changes in HR after different events. Figure 4A shows that there was no statistical difference in the HR of the initial healthy mice (*P* > 0.05). After 7 injections, the HR (Fig. 4B) did not change significantly. After 21 days of placement, the HR (Fig. 4C) of Group A, Group C and Group D decreased significantly (*P* < 0.05). Figure 4D is a recorded echocardiogram and the original images have been in Additional file 1.

#### Changes of the three parameters of echocardiography with the change of days. (7 days for modeling, 21 days for placement)

In order to explore the changes of EF, FS and HR during the modeling process, we added 6 mice to each group repeated the experimental operation and recorded the changes of these three indicators.

**Fig. 5:**
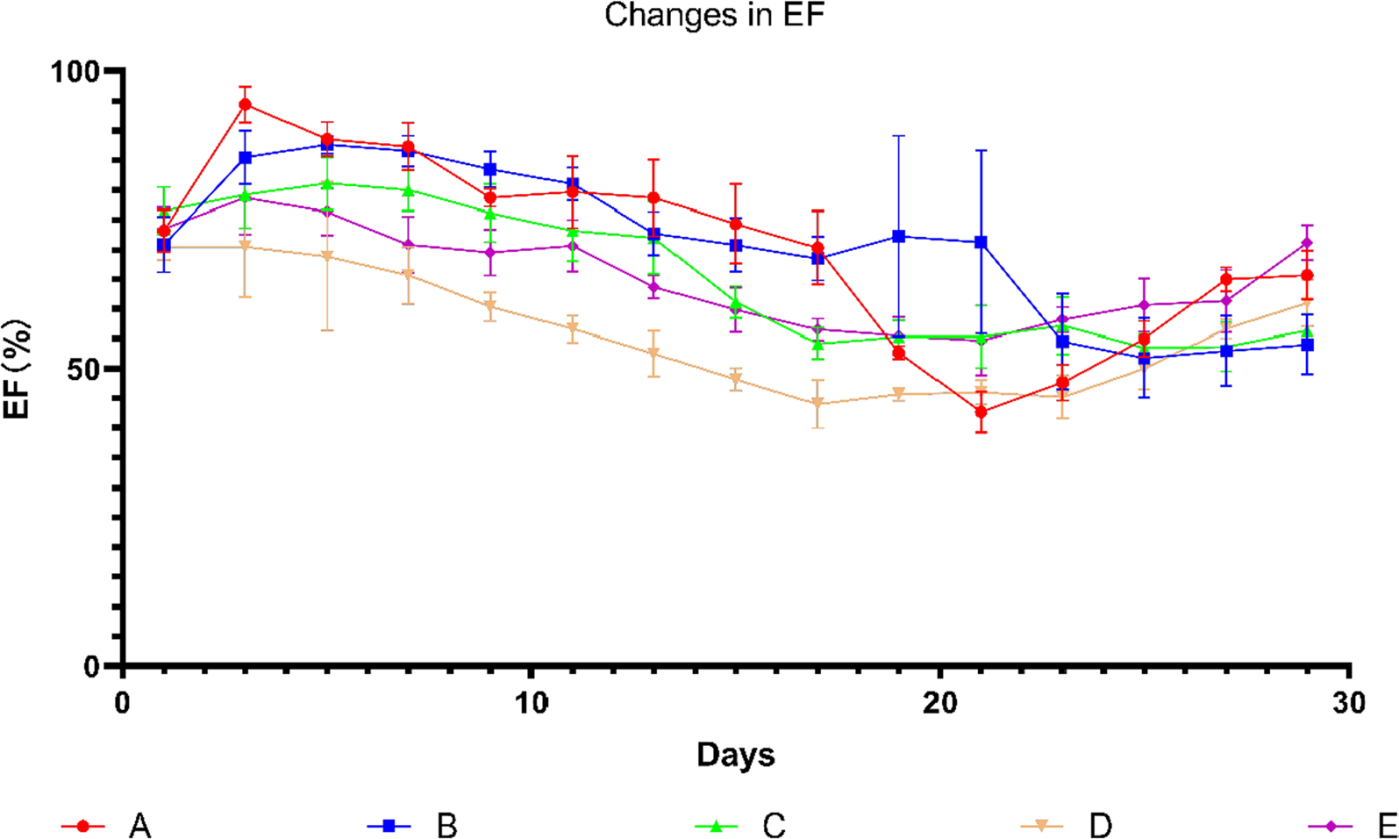
Graph showing changes in EF over days. Data shown are means ± SD; n = 6 in each group. The red line represents Group A, the blue line represents Group B, the green line represents Group C, the yellow line represents Group D, and the purple line represents Group E. Group S was not recorded in this experiment

There was no significant difference in initial EF. Group A and Group B showed significant increase in EF at the initial stage of drug injection, then decreased and finally showed the damage of cardiac function. The EF of Group C and Group D decreased gradually from the initial stage and finally showed the damage of cardiac function. There was no obvious change of EF in Group E and there was no significant difference between the initial EF and the final EF (Fig. 5).

**Fig. 6:**
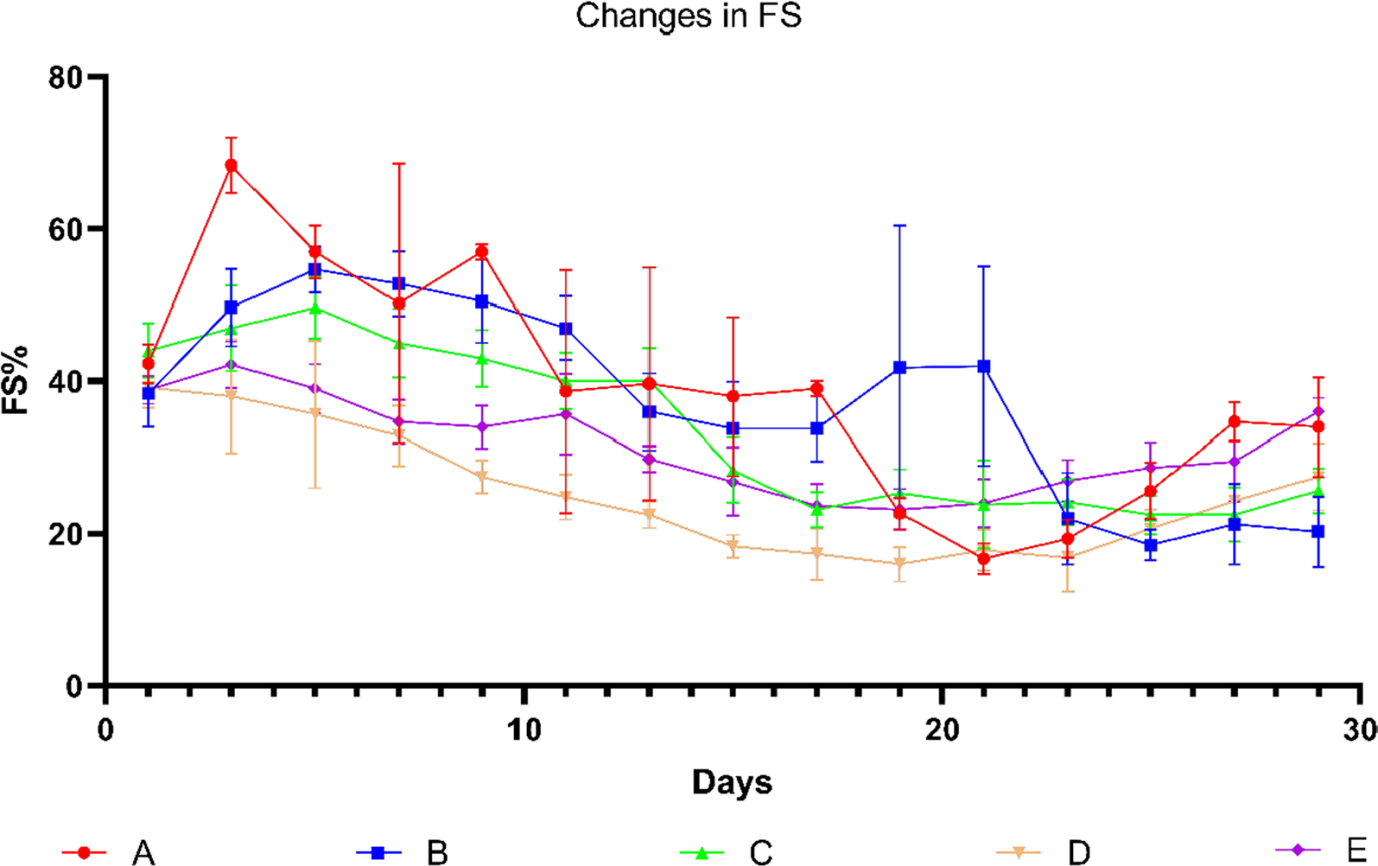
Graph showing changes in FS over days. Data shown are means ± SD; n = 6 in each group. The red line represents Group A, the blue line represents Group B, the green line represents Group C, the yellow line represents Group D, and the purple line represents Group E. Group S was not recorded in this experiment

The changes of FS had no obvious regularity but besides the Group E, others also showed different degrees of cardiac function damage, which was close to the results of EF (Fig. 6).

**Fig. 7:**
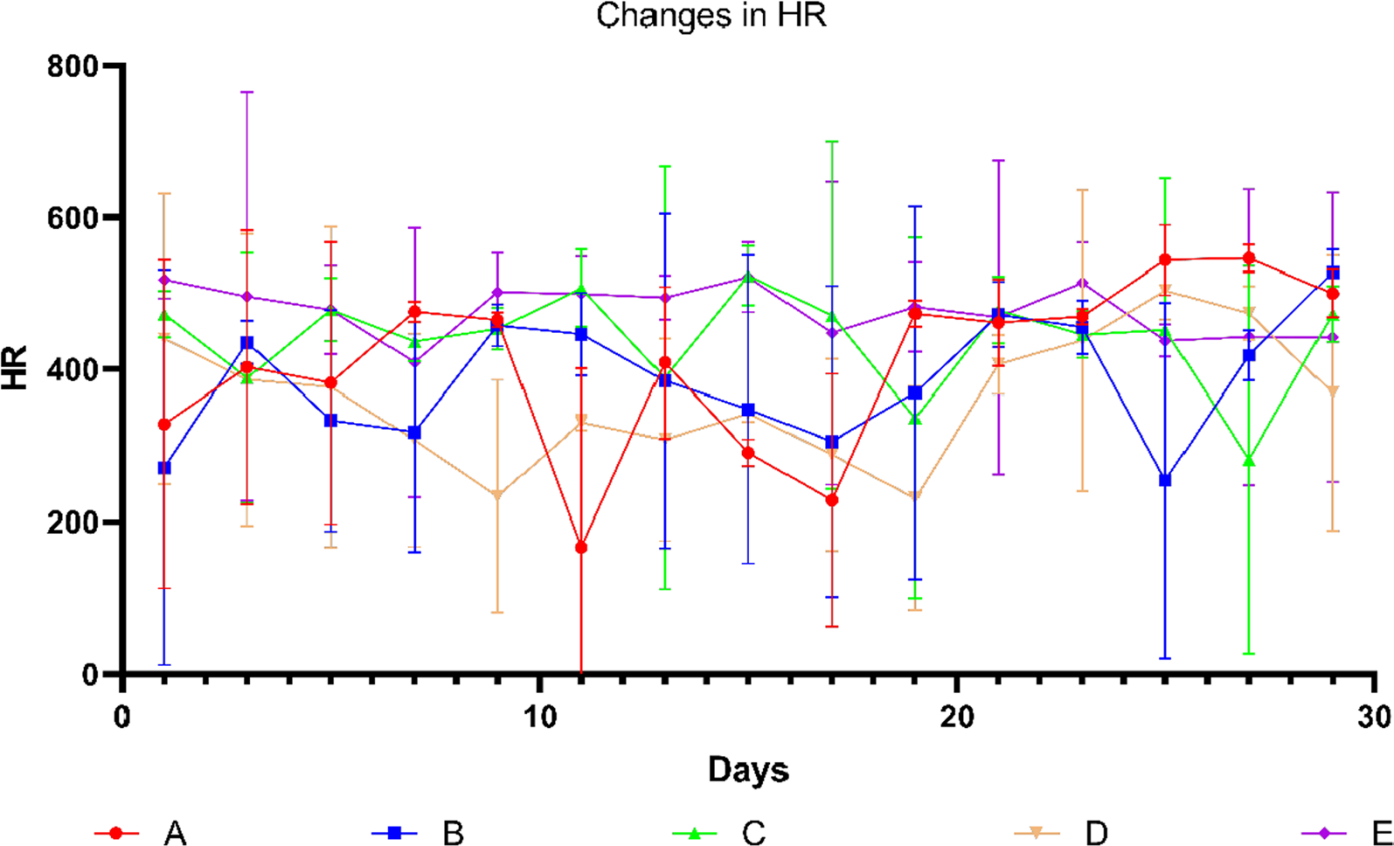
Graph showing changes in EF over days. Data shown are means ± SD; n = 6 in each group. The red line represents Group A, the blue line represents Group B, the green line represents Group C, the yellow line represents Group D, and the purple line represents Group E. Group S was not recorded in this experiment

The change of HR was messy, but we still keep track (Fig. 7).

#### It was found that except for the Group S, the mice in each group had different degrees of BNP and CRP elevation by ELISA

We found by echocardiography that there was no impaired cardiac function in Group E (no significant changes in EF, FS and HR), so Group E was canceled in the following-up study.

**Fig. 8:**
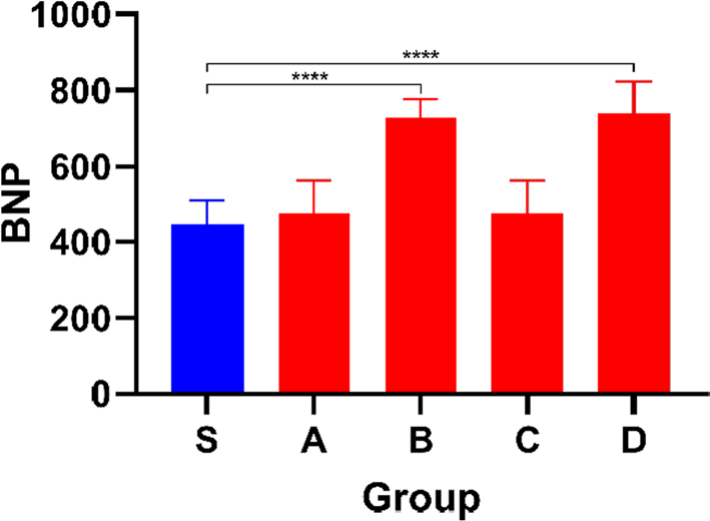
Bar graph showing BNP levels in different groups. Data shown are means ± SD; n = 6 in each group. *P < 0.05, significantly different as indicated

BNP is a key marker for the diagnosis of HF and the detection of BNP is more helpful for us to determine the standard of ISO modelling. BNP was significantly increased in Group S compared with Group B and Group D (*P* < 0.05), which confirmed the occurrence of HF (Fig. 8).

**Fig. 9:**
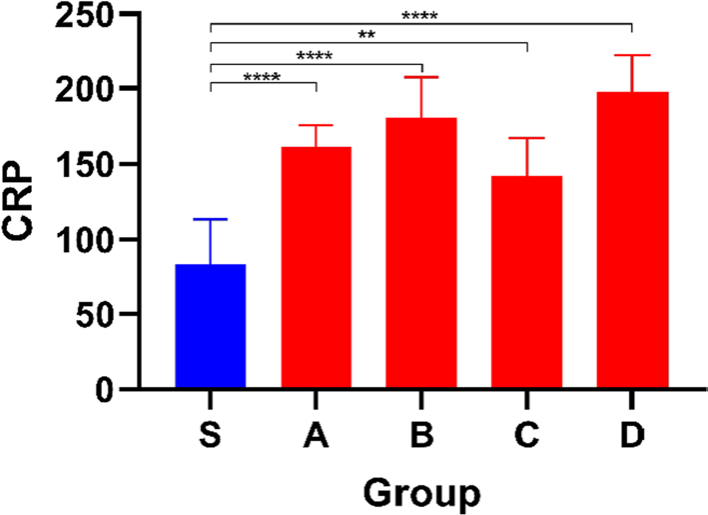
Bar graph showing CRP levels in different groups.Data shown are means ± SD; n = 6 in each group. *P < 0.05, significantly different as indicated

Inflammation plays an important role in the development of HF and we measured C-reactive protein (CRP). Compared with Group S, the CRP of the mice in other groups was significantly increased (*P* < 0.05), which also confirmed the occurrence of inflammatory response (Fig. 9).

### Secondary outcome measures

#### Secondary outcomes included echocardiographically recorded FS

**Fig. 10:**
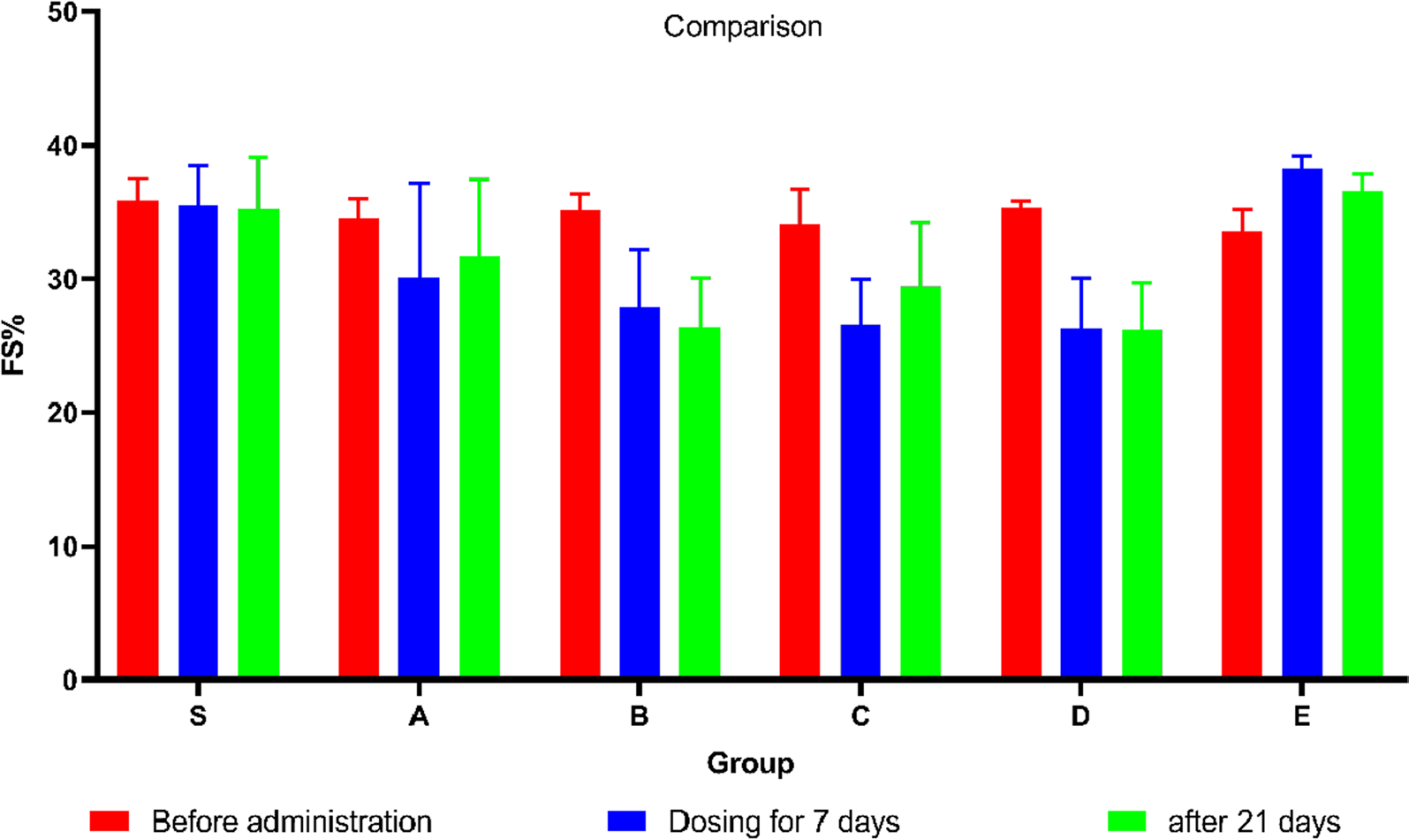
The figure showing the FS before administration (Blue), FS of dosing for 7 days (Red) and FS after 21 days (Green) in groups

**Fig. 11:**
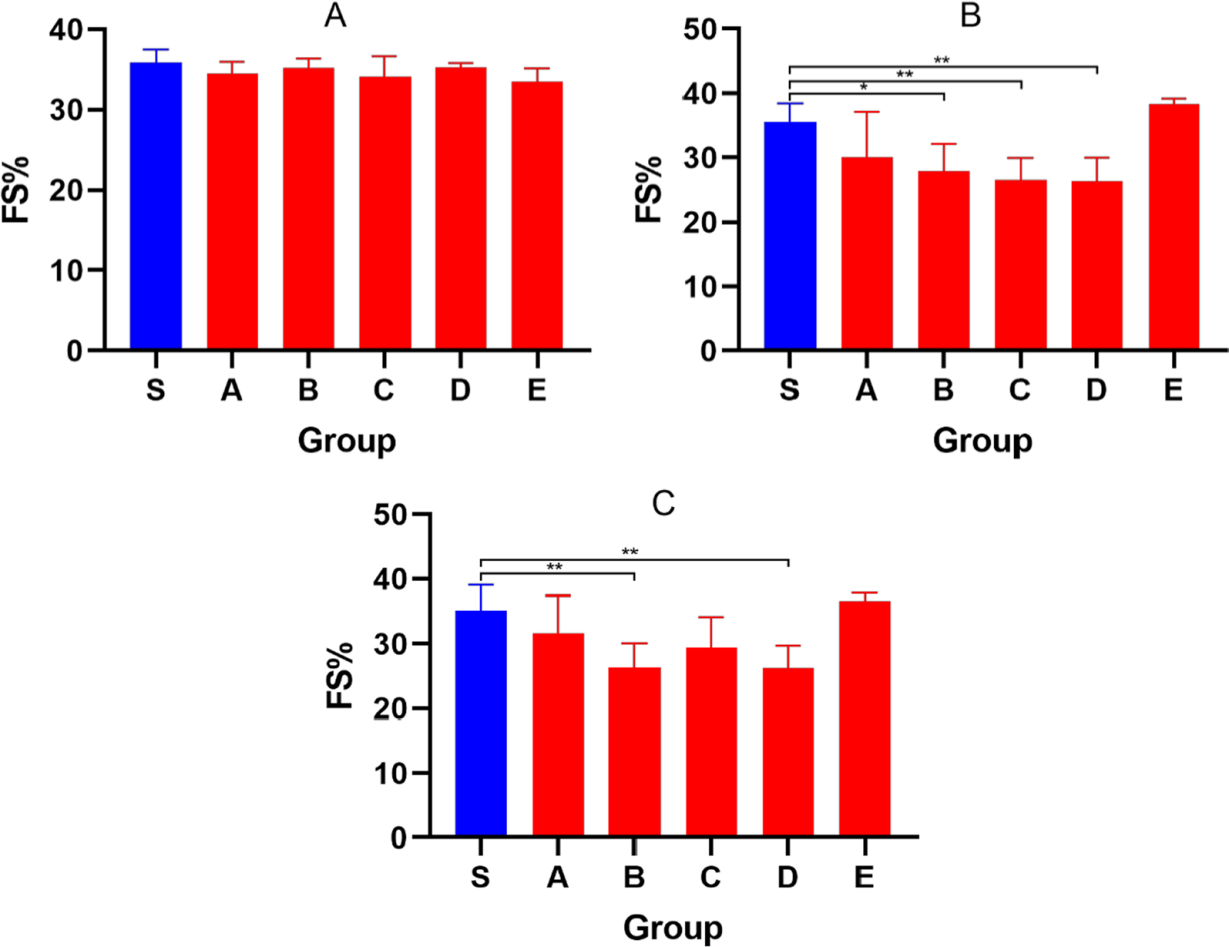
Bar graphs showing the FS before administration(A), FS after dosing for 7 days(B), FS after 21 days(C). Data shown are means ± SD; n = 6 in each group. *P < 0.05, significantly different as indicated

FS can reflect changes in cardiac function too. Figure 10 shows the changes in FS after different events. Figure 11A shows that there was no statistical difference in the FS of initial healthy mice (*P* > 0.05). After 7 injections, the FS (Fig. 11 B) of Group B, Group C and Group D decreased significantly (*P* < 0.05). After 21 days of placement, the FS (Fig. 11C) of Group B and Group D was still significantly different from that of Group S (*P* < 0.05).

#### After injection of ISO, inflammatory cells increased in the heart tissue of mice in each group

**Fig. 12:**
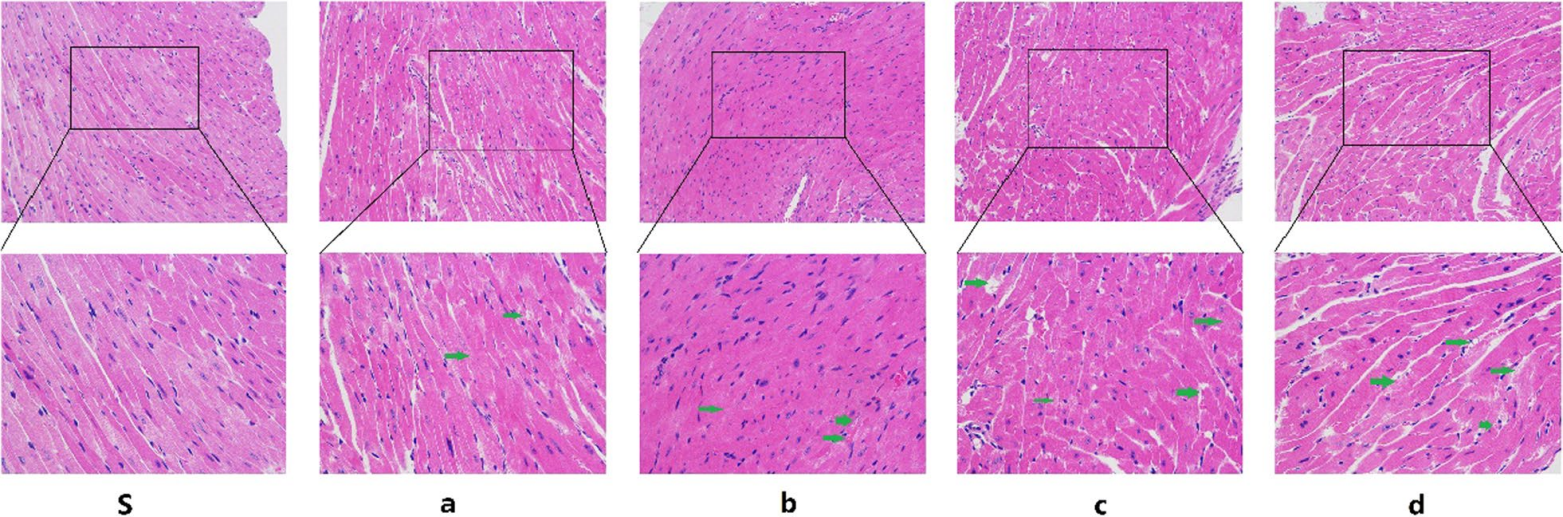
Representative images of HE staining showing changes in inflammatory cells in cardiac sections from each group of mice. Green arrows indicate exceptions

HE staining results revealed the degree of myocardial tissue in mice models. Through HE staining, we observed myofibrolysis and nuclear enlargement in the other groups compared with Group S (Fig. 12).

## Discussion

CHF causes myocardial tissue injury and cardiac systolic and diastolic dysfunction due to a variety of reasons [29], but it is not limited to myocardial infarction, hypertension, myocarditis and so on [30]. All studies on heart failure require an appropriate heart failure model. The ideal model should be able to reproduce all aspects of the progression of naturally-occurring congestive heart failure [31]. The CHF model protocol of ISO‐induced mice has the advantages of simplicity and easy operation but it is difficult to replicate for the sake of ambiguous administration dose and nonuniform modeling time. Our study aims to establish a stable and reliable CHF model that meets the experimental purpose to provide a basic choice of models in practical studies, thus to facilitate the development of new treatment strategies for patients with heart failure.

In this study, the criteria of model formation and the related indexes analysis were clarified through studying the previous methods of ISO preparation of C57BL / 6 mice chronic heart failure model and performing group experiments that explore the models of different ISO doses, combining with the current conditions and partial experience of our own laboratory and the characteristics of the CHF model preparation protocol [32]. We found that Group A, Group B, Group C and group D can all cause CHF but the mortality rate of Group A and Group B is higher and the mice with higher doses will have skin ulceration and other phenomena that may affect the following research. Group D has a high success rate of modeling and should be the recommended dose for CHF mouse model.

To establish a recommended and stable animal model of heart failure is of great significance for relevant researches. Over the past few decades, many small animal models have been generated to mimic various pathological mechanisms resulting in heart failure. Despite some limitations, these animal models have greatly advanced our understanding of the pathogenesis of heart failure in etiology and have paved the way for understanding the underlying mechanisms and the development of successful therapies [33]. Although close to humans, the cardiac structure of large animals has relatively few applications due to the high modeling cost and complicated operation [34]. Relatively, small animal models are more commonly used when performing relevant medical and pharmacological studies [33].C57BL/6 mice are commonly used experimental animals for related medical and pharmacological research. They have become one of the most commonly used animal models of heart failure because of their short time of reproduction, easy genetic modification, good stability and low cost [30].

ISO has been widely reported to cause heart failure in animals [35, 36] and the ISO induced CHF model is applicable to various inbred strains of mice [37, 38]. Literature reports that the ISO induced myocardial injury model has been widely used to research the beneficial effects of drugs on cardiac dysfunction [39], playing a crucial role in the pathogenesis of HF [40].

The injection doses were divided into three intervals: 30–10 mg/kg, 10–120 mg/kg, 150–400 mg/kg [41]. The first type of long-term intervention resulted in myocardial hypertrophy, causing myocardial overload and increased mortality. Up to 80–90% of prolonged ISO access resulted in advanced hypertrophy characterized by pathological hypertrophy with extensive confluent cardiomyopathy. The second dose resulted in changes in cardiomyocyte energy metabolism. Injection of large doses of ISO resulted in acute myocardial injury, similar to acute myocardial infarction but with a higher mortality rate.

The pathophysiological and morphological abnormalities produced in experimental models of ISO induced CHF are comparable to those occurring in humans. Experimental animal models established by ISO, including pathological myocardial injury, myocardial infarction, cardiac hypertrophy and heart failure, are beneficial to our understanding β- Pathological changes and pathological mechanisms under adrenergic receptor stimulation and finding the best way to treat sympathetic overactivation.

Chronic stimulation of ISO to G-protein-coupled ß-adrenergic receptor causes cardiomyocyte hypertrophy and fibrosis in mice and rats. It resembles the development of progressive heart failure aroused by cardiac specific overexpression of the β1-ADR in mice, suggesting that mice can model aspects of the pathogenesis of CHF. Underlying pathogenic mechanisms of the disease can be explained by the model [33]. The ISO induced CHF model is non-invasive, easy to operate and highly reproducible and can well reflect the natural pathological process of CHF. Though it is simple to operate and easy to handle, it is affected by different batches of animals, different drug lots, different routes of administration. Pre-experiments must be performed before testing to determine the final experimental protocol.

CHF models are usually prepared using favorable surgical procedures, chemical induction, genetic modification, genetic techniques and hypertension induction. Since the most important part of the etiology of CHF is ischemic origin [42], most studies use myocardial infarction models to explore CHF, usually by ligating the left anterior descending artery, resulting in CHF 4 weeks after operation [43]. This model is an acute ischemic injury model [44], generally used to evaluate the remodeling of cells and extracellular matrix after myocardial infarction but not suitable for the long-term exploration of neuroendocrine function [31].

ISO is a synthetic catecholamine and non-selective β- Adrenergic agonist [45], which agonizes the heart β1 receptor to exerts a positive effect on the myocardium, leading to a marked drop in diastolic blood pressure caused by intense vasodilation and in turn giving rise to coronary hypoperfusion, persistently producing cardiac dysfunction and left ventricular dilation [46]. Studies have shown that adrenergic receptors are involved in regulating physiological and pathological processes in the myocardium and their increasing drive plays an essential role in compensating for the decline in cardiac function [47].

β-Adrenergic receptors (β-ARS) chronic hyperactivity of signal transduction is the interface between sympathetic nerve fibers and deterioration of cardiac function [48]. As an independent risk factor for cardiovascular mortality [49–51], cardiac fibrosis is an adaptive remodeling process during cardiac injury [52],whose underlying reasons include mechanical stress, inflammation, ischemia and neurohormonal overactivation. It is characterized by the production of excessive extracellular matrix (ECM) due to the accumulation of inflammatory cells and activated cardiac fibroblasts (CFS). Many lines of evidence indicate that the progression of heart failure manifested by ventricular diastolic and systolic dysfunction is characterized by a marked cardiac hypertrophic and myocardial fibrotic response [53–56] and ISO can stimulate adrenaline and promote cardiac inflammation and fibrosis, causing decompensation and left ventricular remodeling featured by cell death and the generation of inflammation [57], contributing to the development of myocardial injury and cardiac remodeling models [58, 59].

However, this study has some shortcomings. We did not experimentally compare the differences in CHF caused by subcutaneous injection of ISO and other methods, which do not fully reflect the characteristics of each model. We only divided injection doses into 5 groups, so there may be better modeling doses. Last but not least, ISO-induced myocardial injury is usually a variable method that some animals develop more, some less injury. We did not consider this issue, we will continue to explore improvements in follow-up research.

## Conclusion

Our research finally found that the HFrEF mice model created by injection at a dose of 100 mg/kg for 7 days was the most suitable and a relatively stable chronic heart failure model could be obtained by placing it for 21 days. Our study aimed to confirm the criteria for a mice model of CHF and will continue to explore the development and treatment mechanisms of CHF in subsequent studies.

## Supplementary Information

**Additional file 1**. Recorded echocardiogram and the original images.

## Data Availability

The datasets used and/or analyzed during the current study are available from the corresponding author on reasonable request. The data that support the findings of this study are available from the corresponding author upon reasonable request. Some data may not be made available because of privacy or ethical restrictions.

## Abbreviations

HF: Heart failure
CHF: Chronic heart failure
BNP: Brain natriuretic peptide
CRP: C-reactive protein
TAC: Transverse aortic coarctation
LV: Left ventricular
EF: Ejection fraction
FS: Fraction shortening
HR: Heart rate
ISO: Isoproterenol
