# Supplementary material for: Effect of injection of different doses of isoproterenol on the hearts of mice
Source: BMC Cardiovasc Disord. 2022 Sep 12;22:409. doi: 10.1186/s12872-022-02852-x (PMC9469628; doi:10.1186/s12872-022-02852-x)
Supplement: Supplementary file 1 — Additional file 1. Recorded echocardiogram and the original images. [file 12872_2022_2852_MOESM1_ESM.docx]

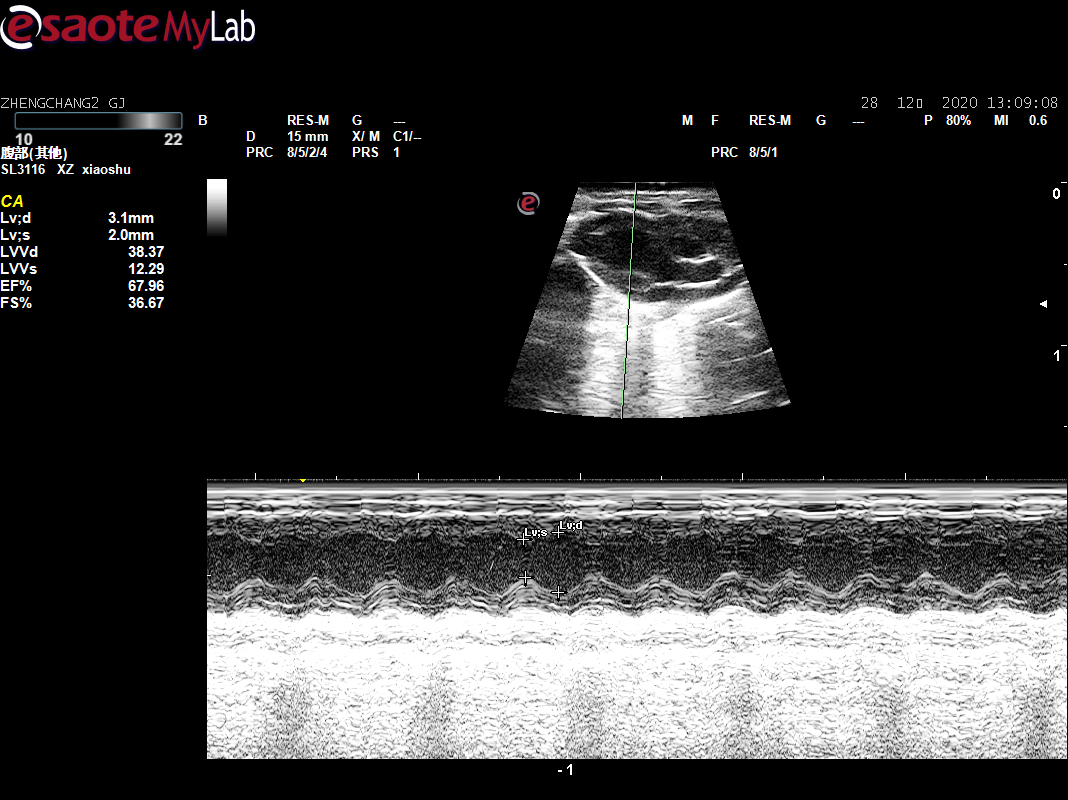


The picture above shows the FS and EF of Group S, before administration


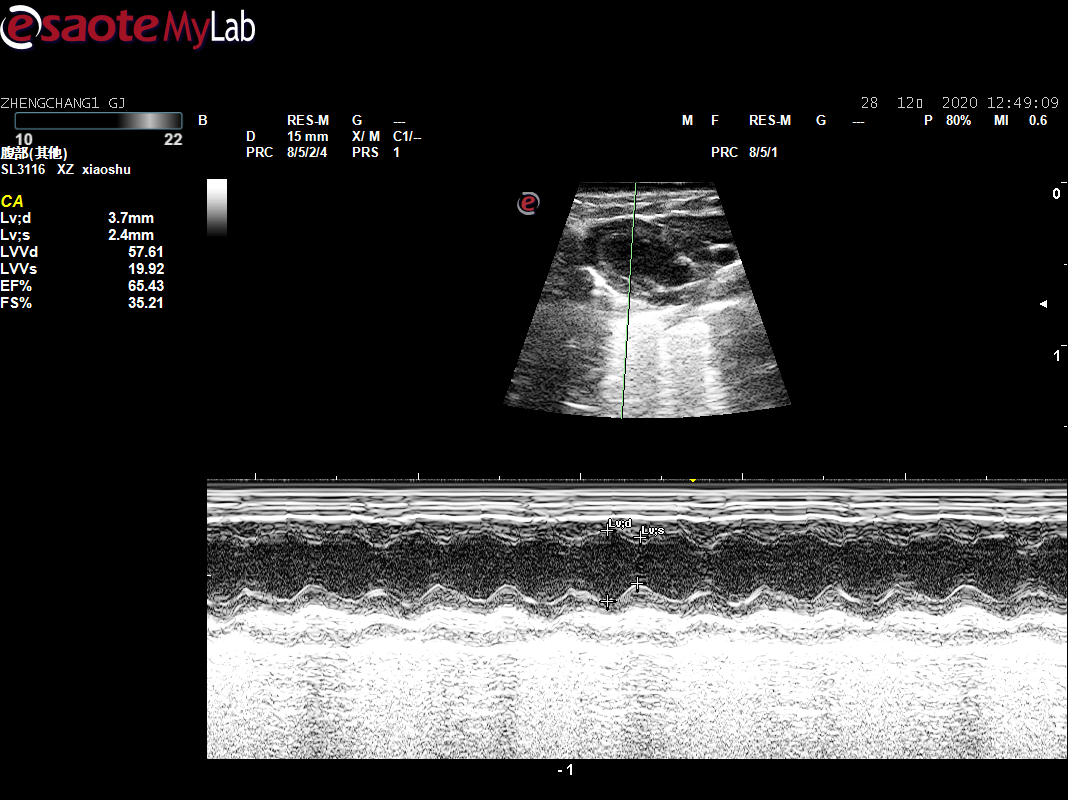


The picture above shows the FS and EF of Group A, before administration


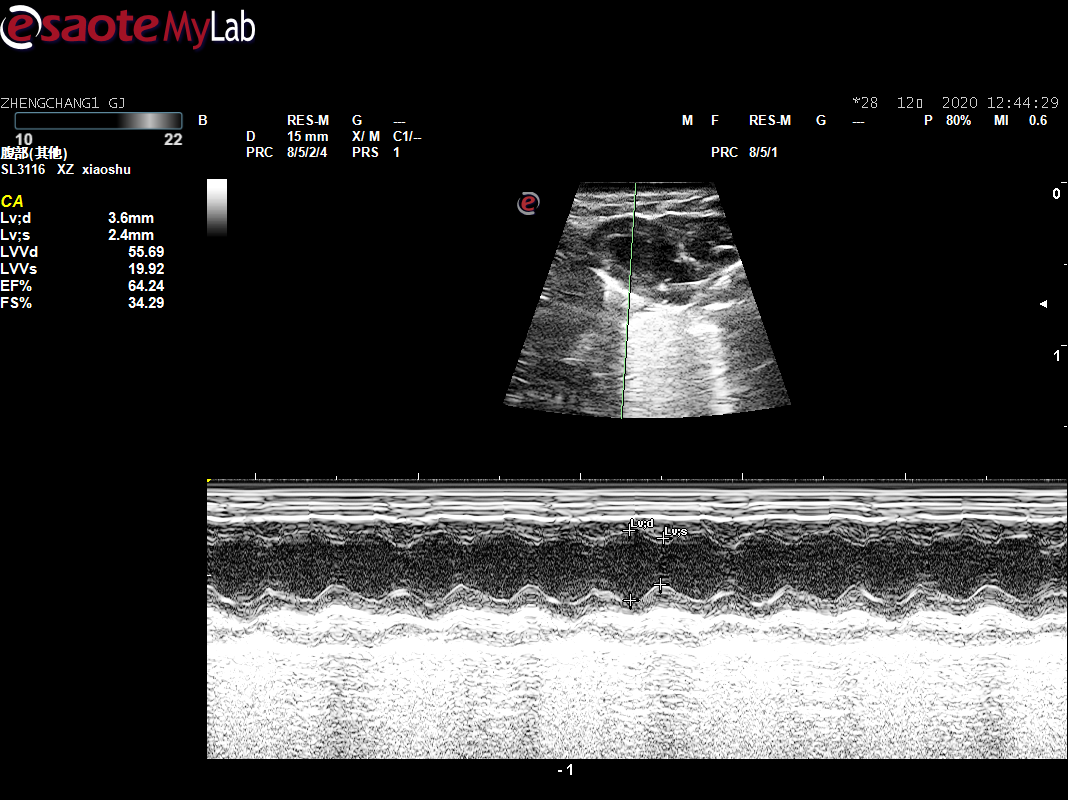


The picture above shows the FS and EF of Group B, before administration


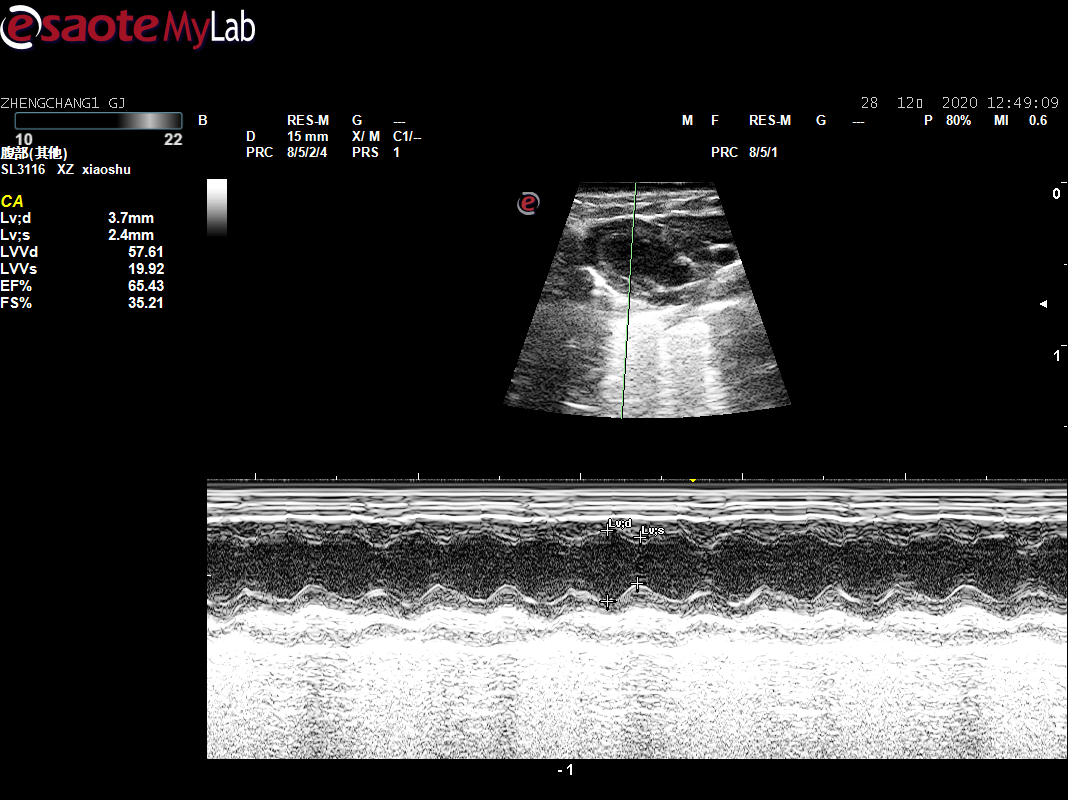


The picture above shows the FS and EF of Group C, before administration


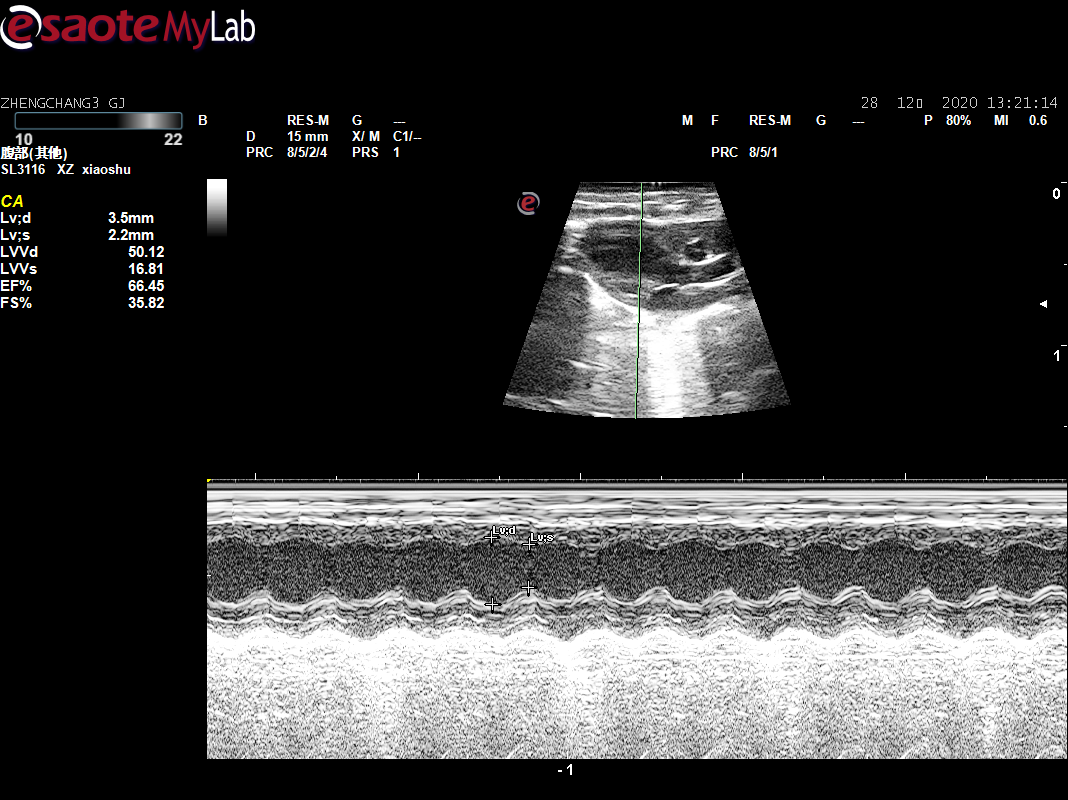


The picture above shows the FS and EF of Group D, before administration


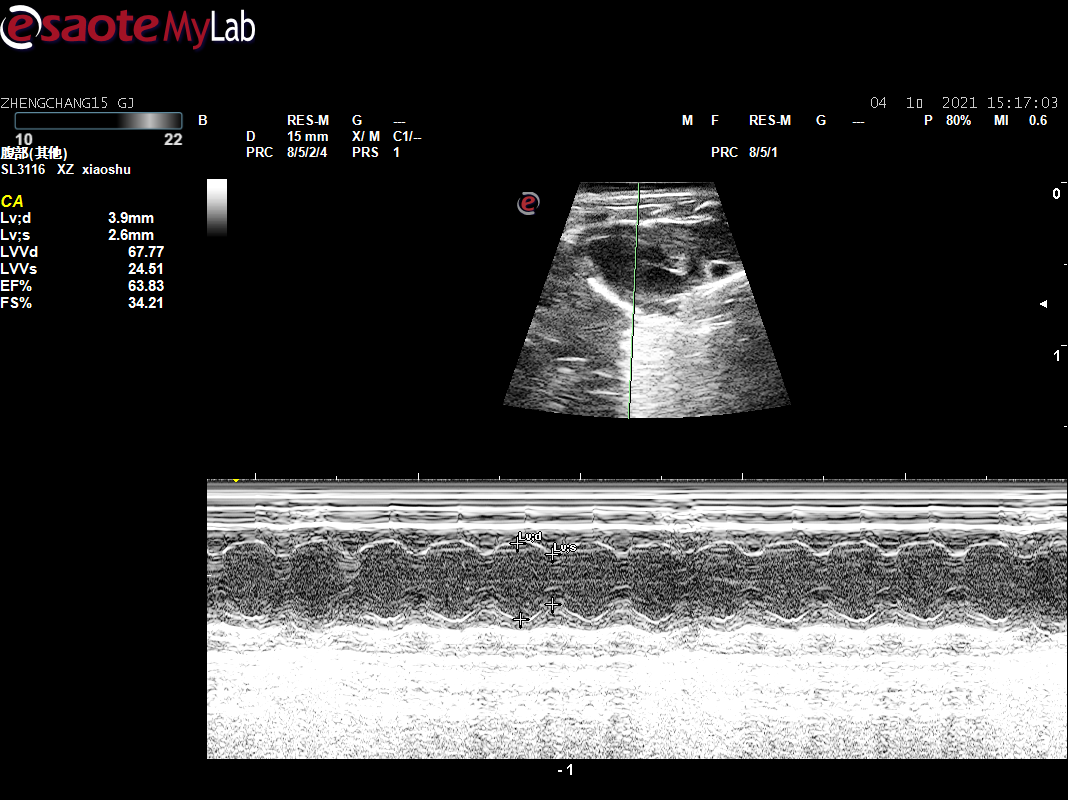


The picture above shows the FS and EF of Group E, before administration


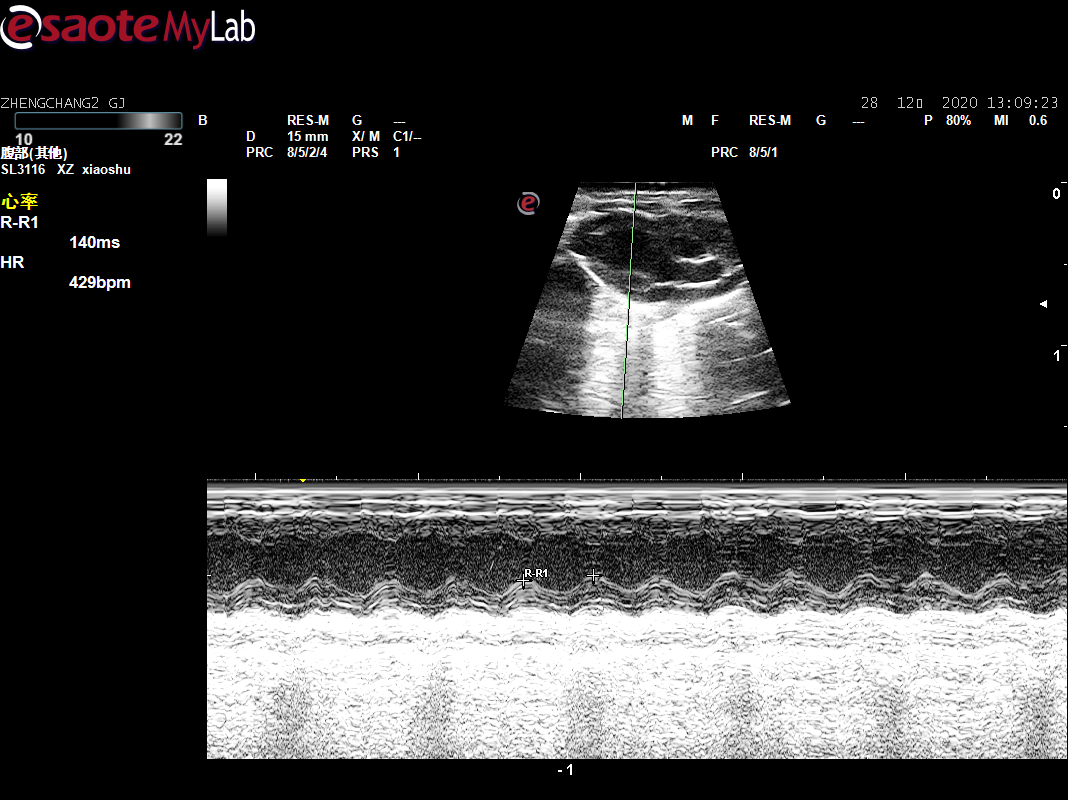


The picture above shows the HR of Group S, before administration


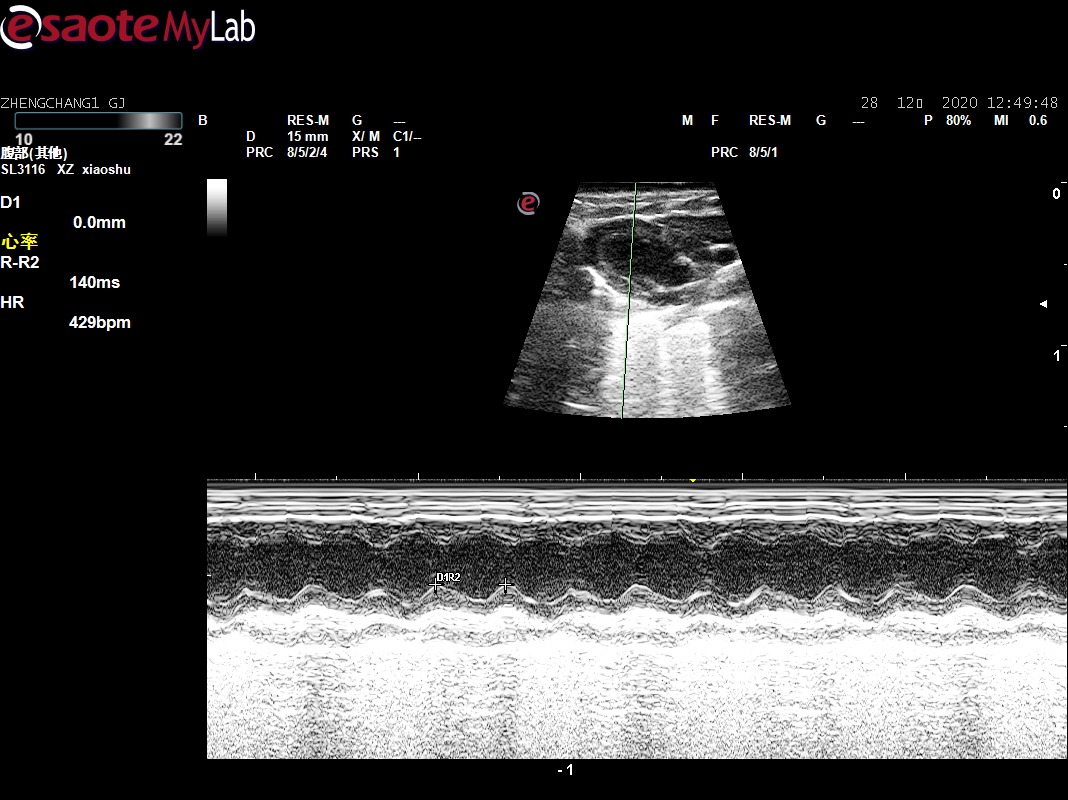


The picture above shows the HR of Group A, before administration


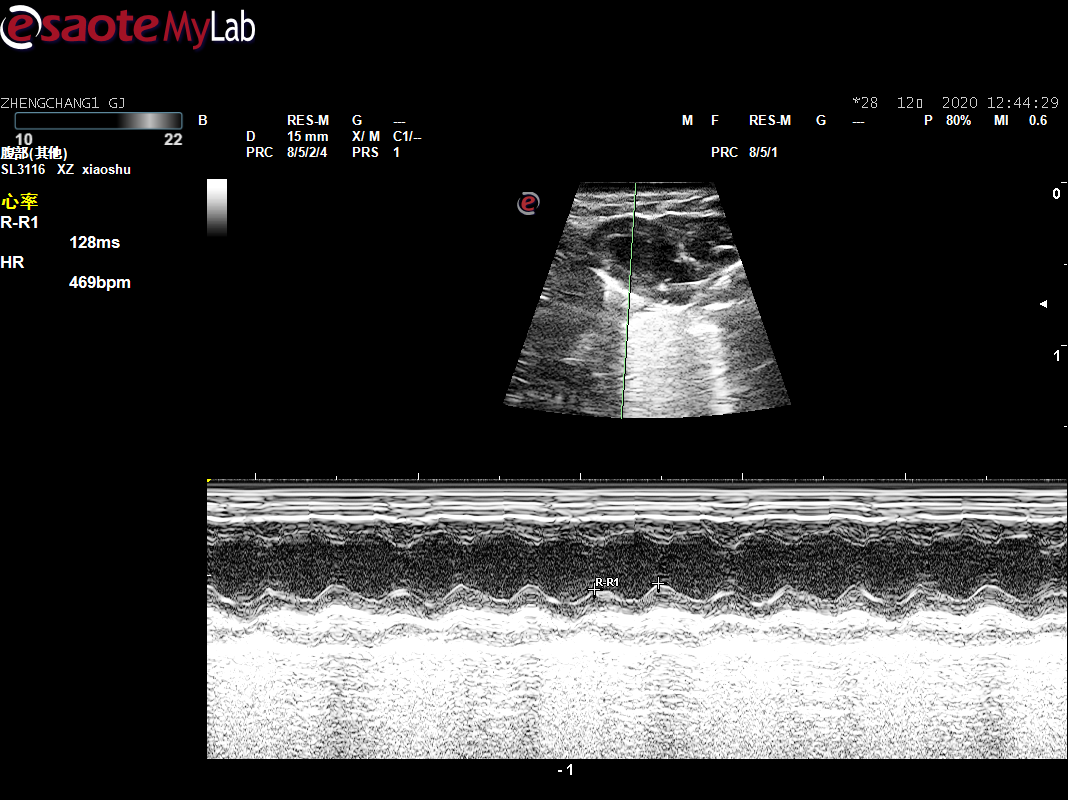


The picture above shows the HR of Group B, before administration


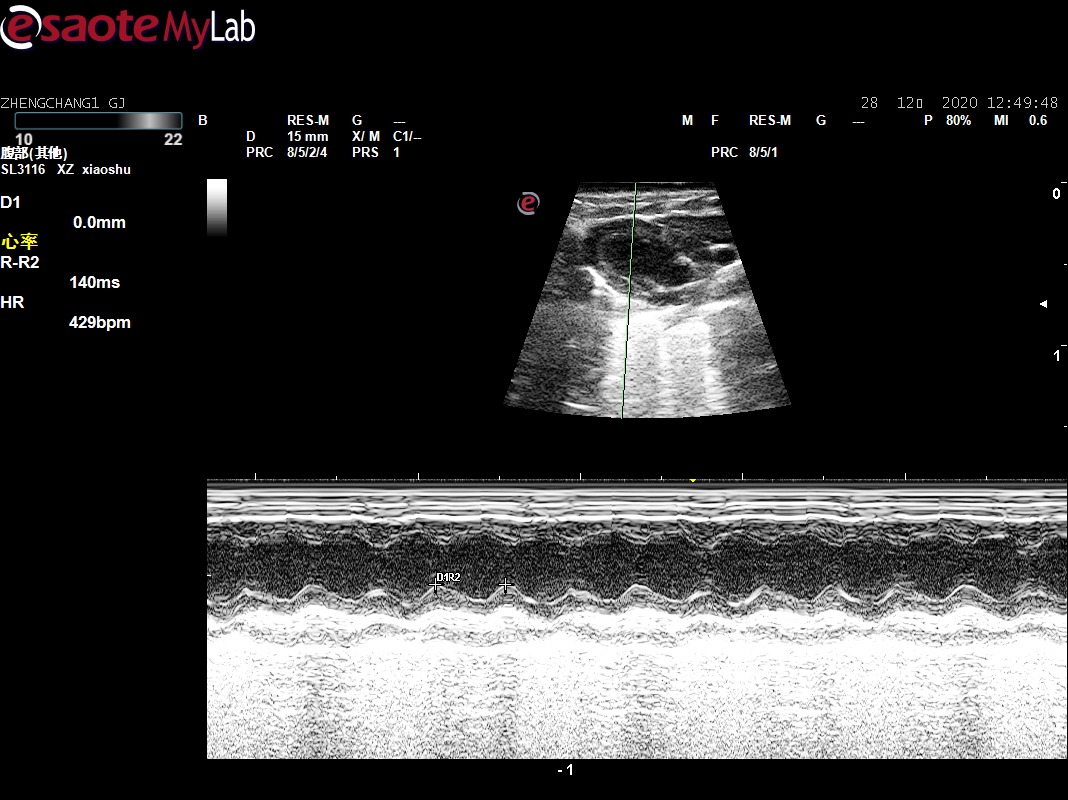


The picture above shows the HR of Group C, before administration


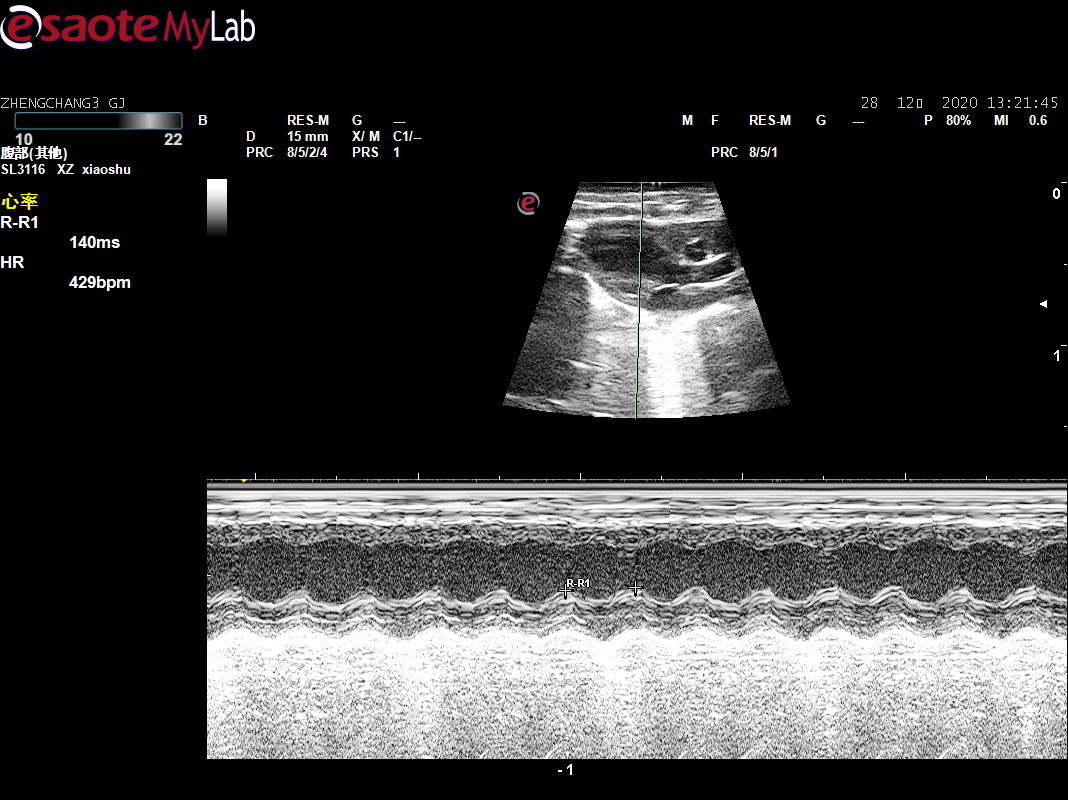


The picture above shows the HR of Group D, before administration


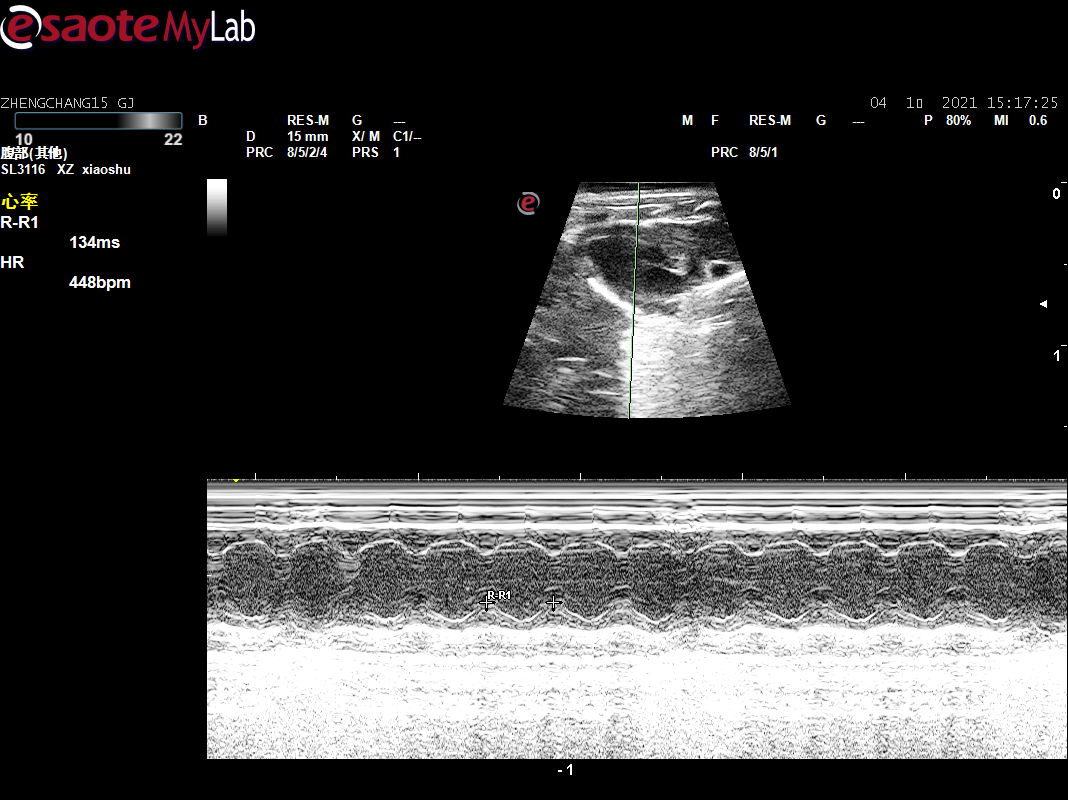


The picture above shows the HR of Group E, before administration


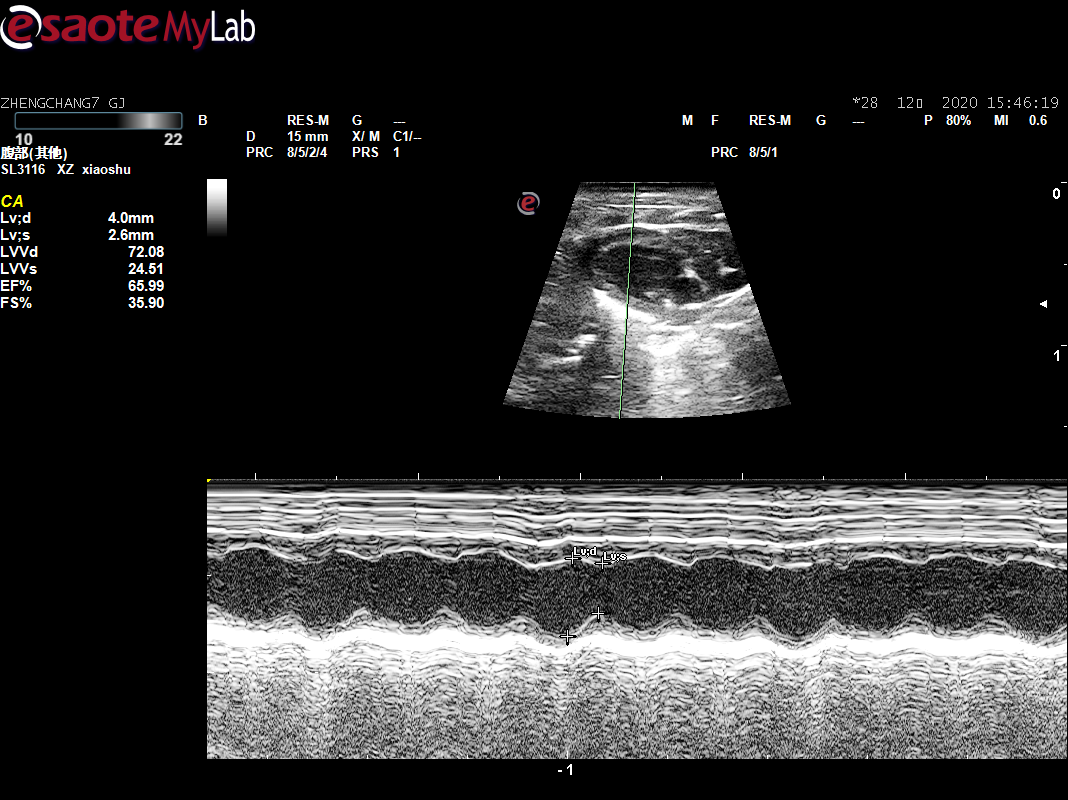


The picture above shows the FS and EF of Group S, dosing for 7 days


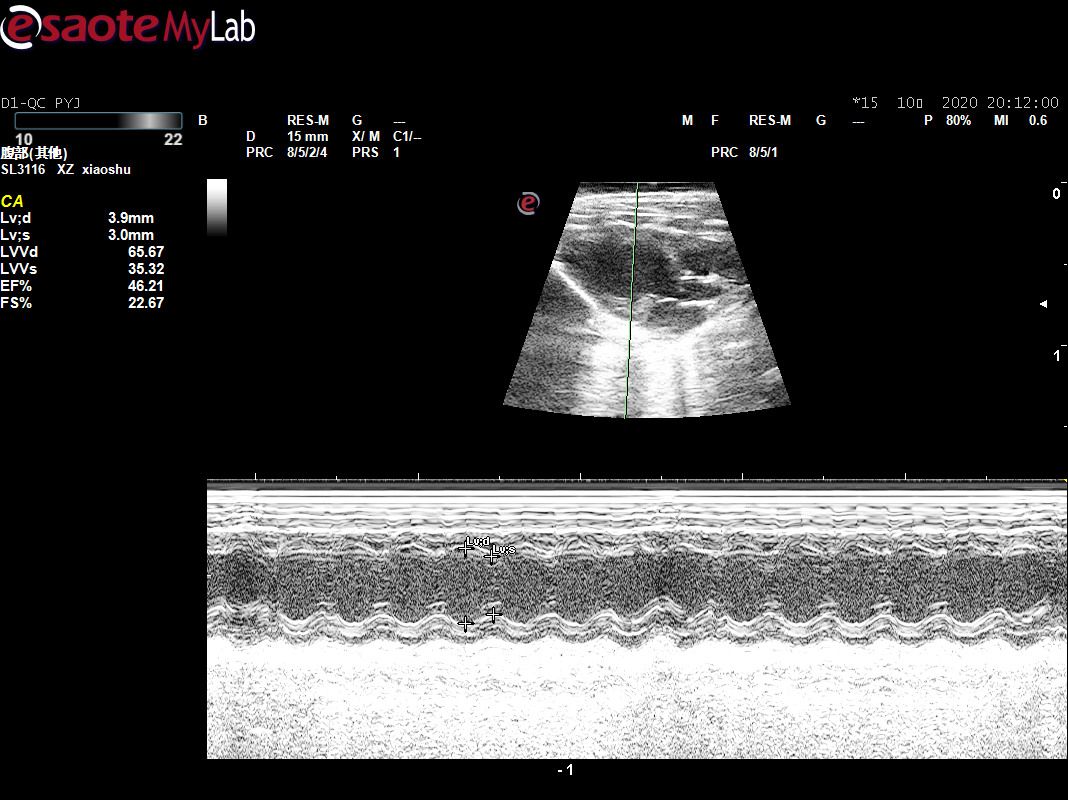


The picture above shows the FS and EF of Group A, dosing for 7 days


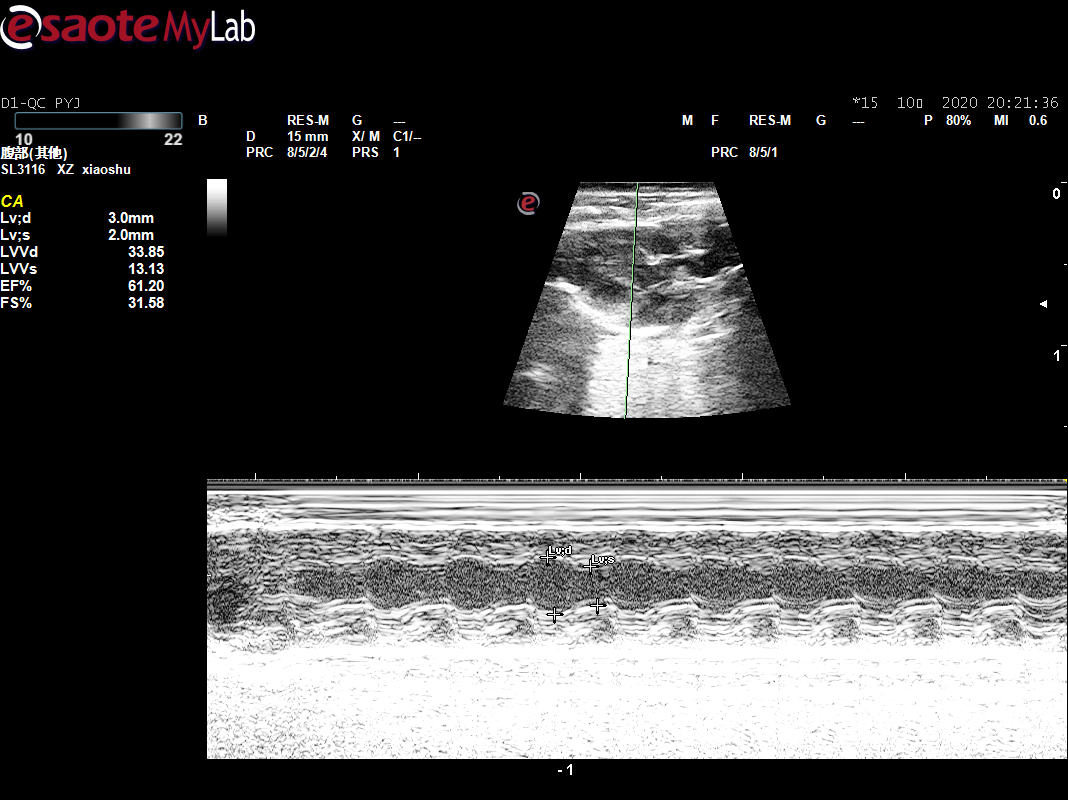


The picture above shows the FS and EF of Group B, dosing for 7 days


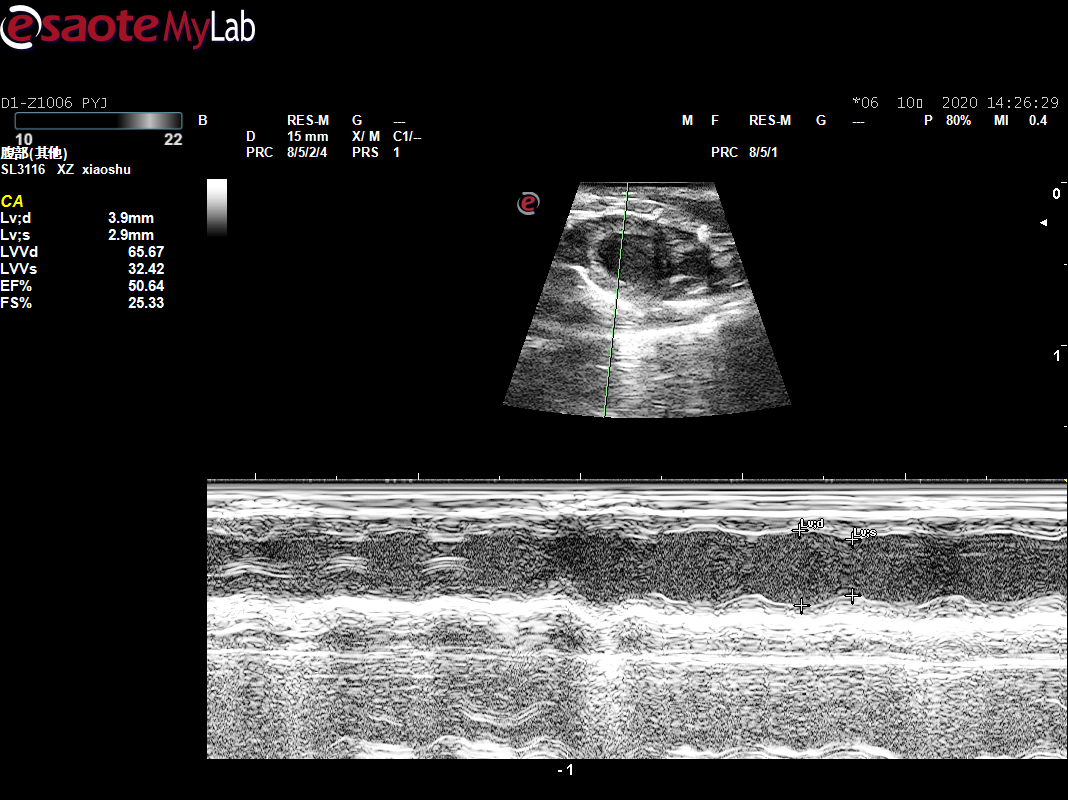


The picture above shows the FS and EF of Group C, dosing for 7 days


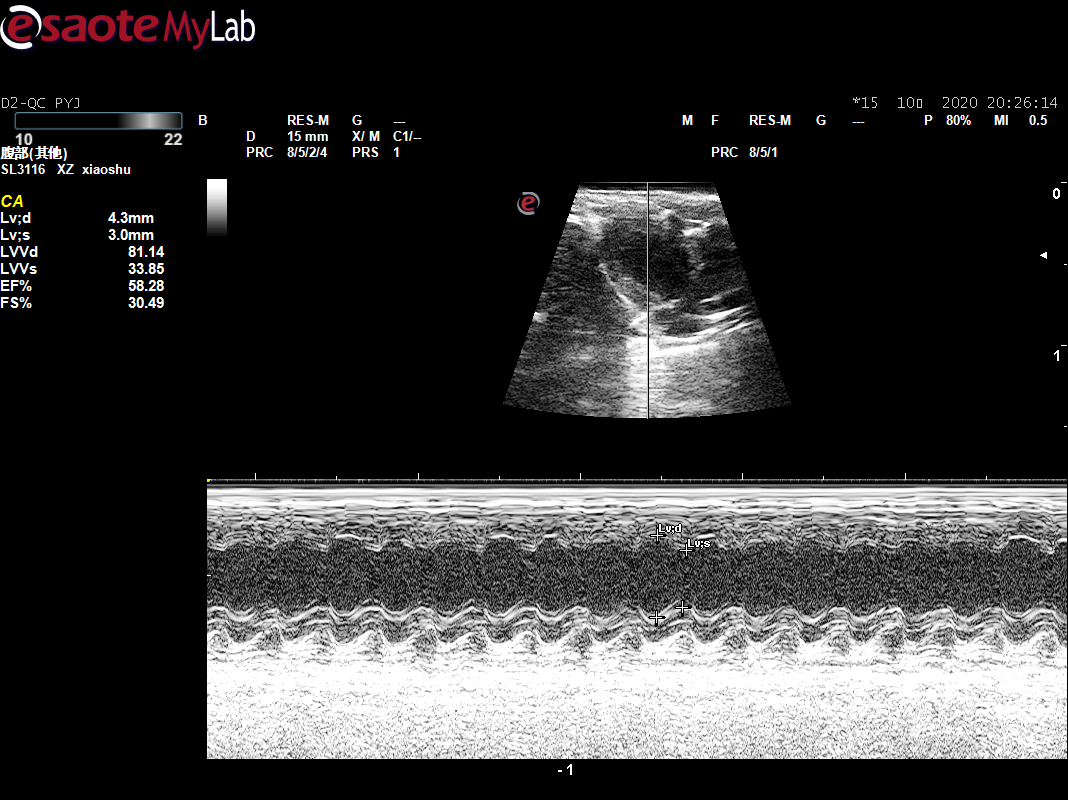


The picture above shows the FS and EF of Group D, dosing for 7 days


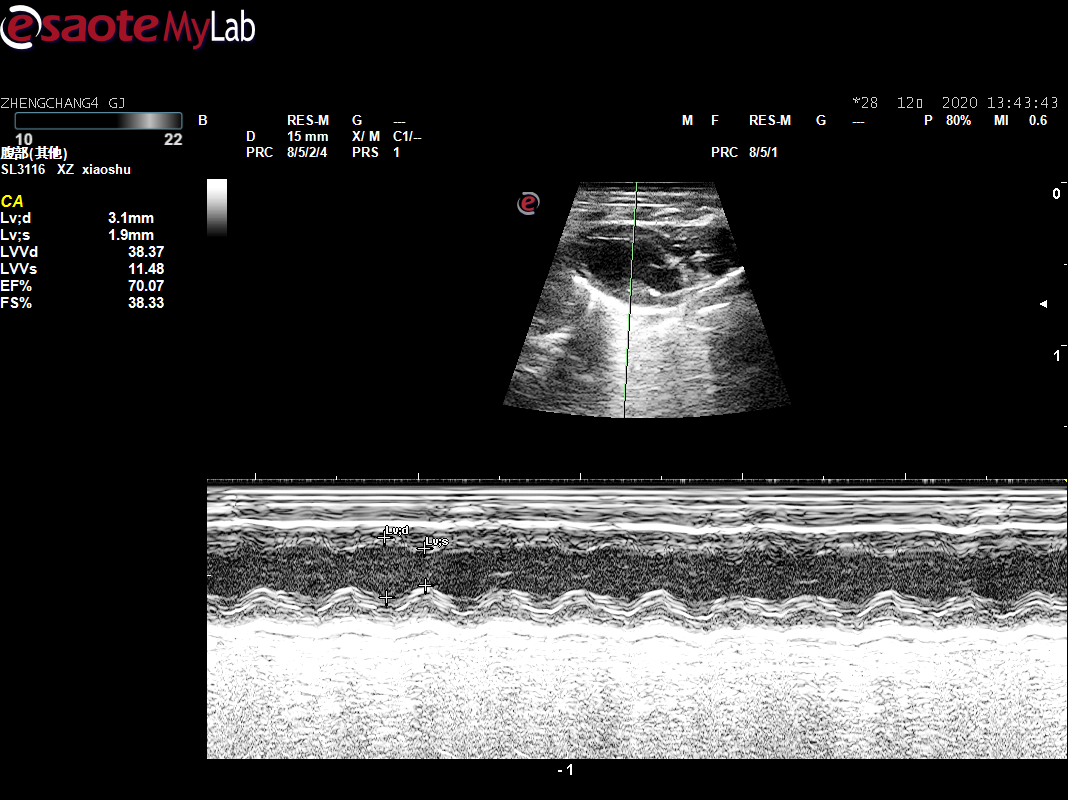


The picture above shows the FS and EF of Group E, dosing for 7 days


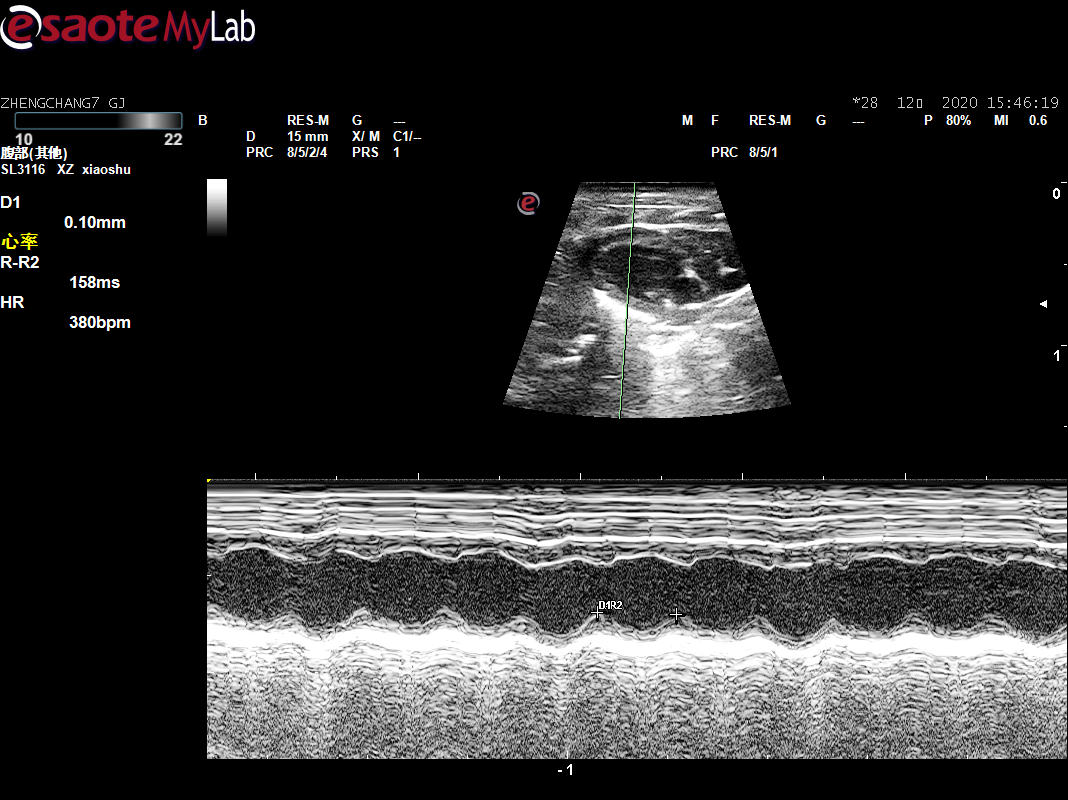


The picture above shows the HR of Group S, dosing for 7 days


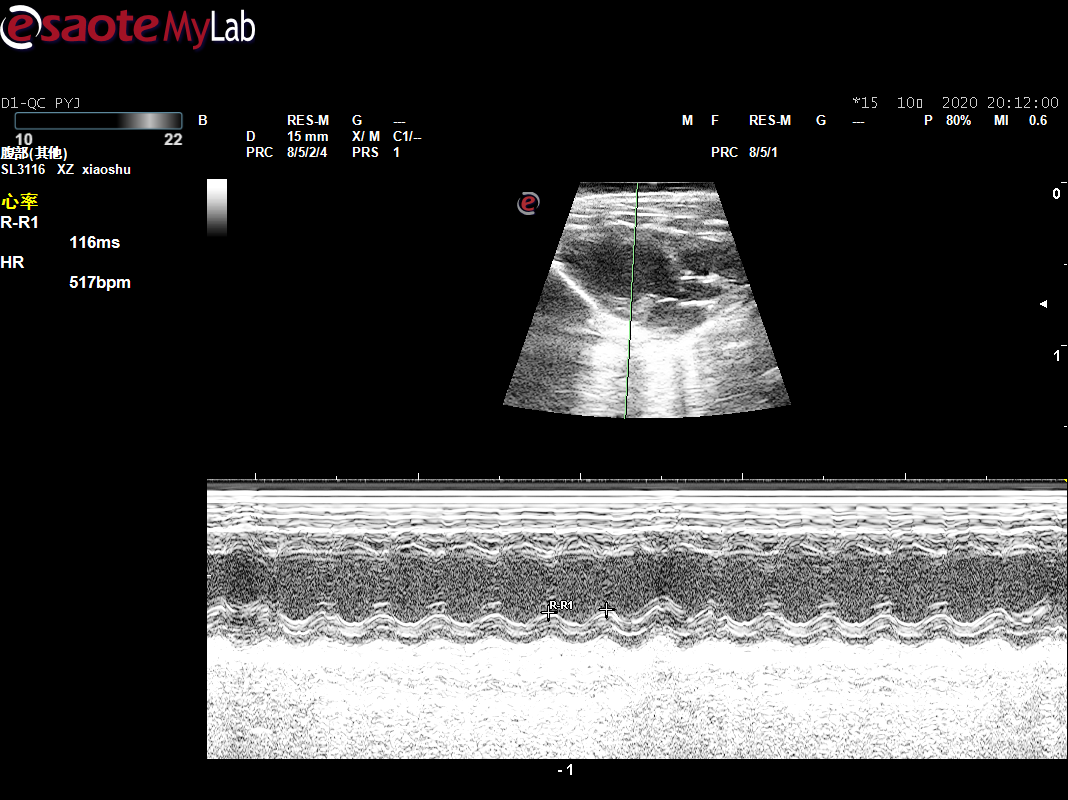


The picture above shows the HR of Group A, dosing for 7 days


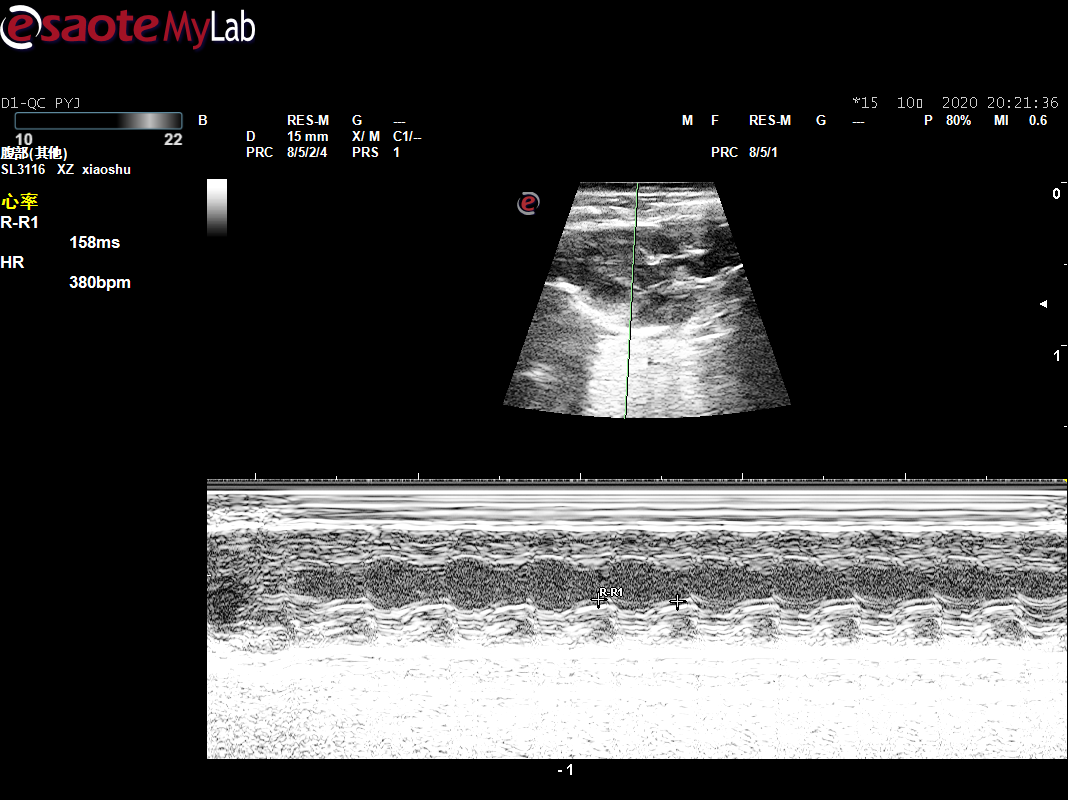


The picture above shows the HR of Group B, dosing for 7 days


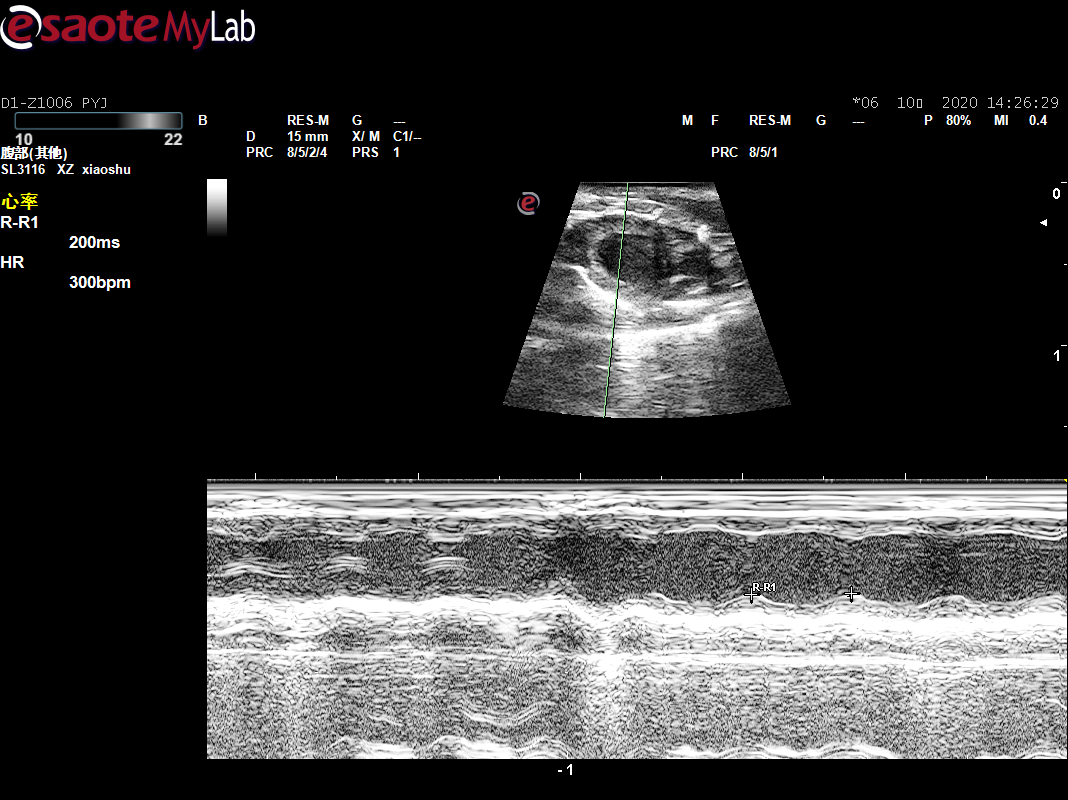


The picture above shows the HR of Group C, dosing for 7 days


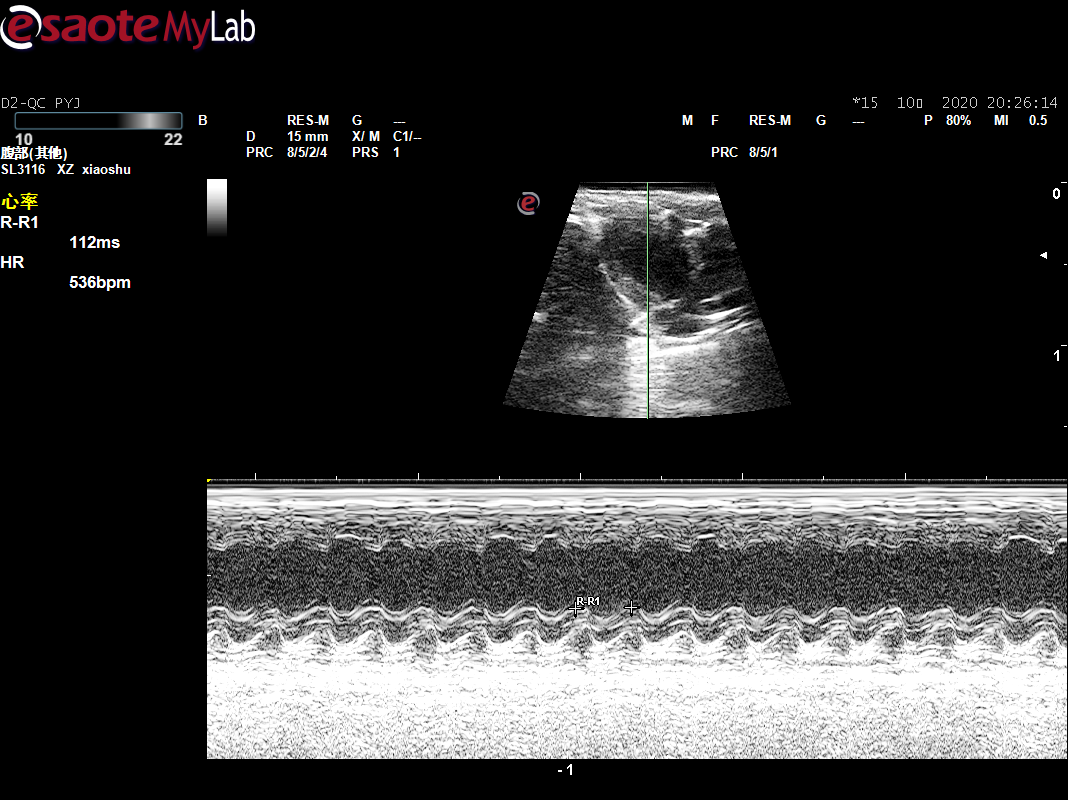


The picture above shows the HR of Group D, dosing for 7 days


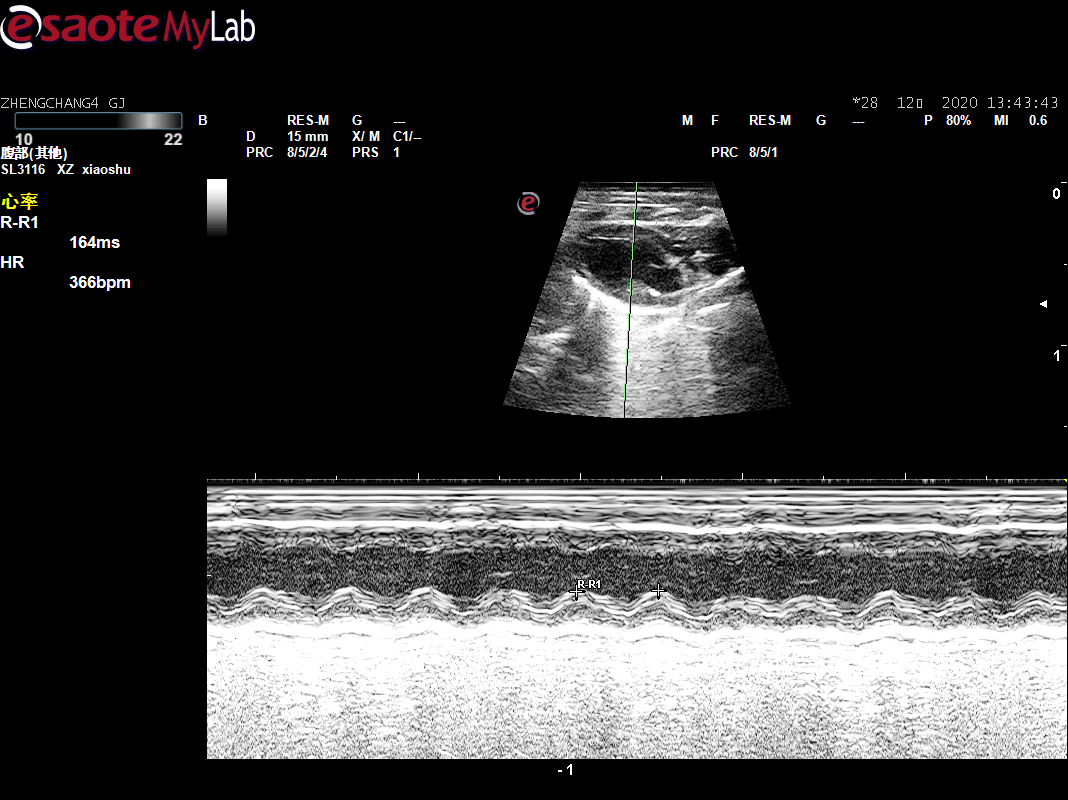


The picture above shows the HR of Group E, dosing for 7 days


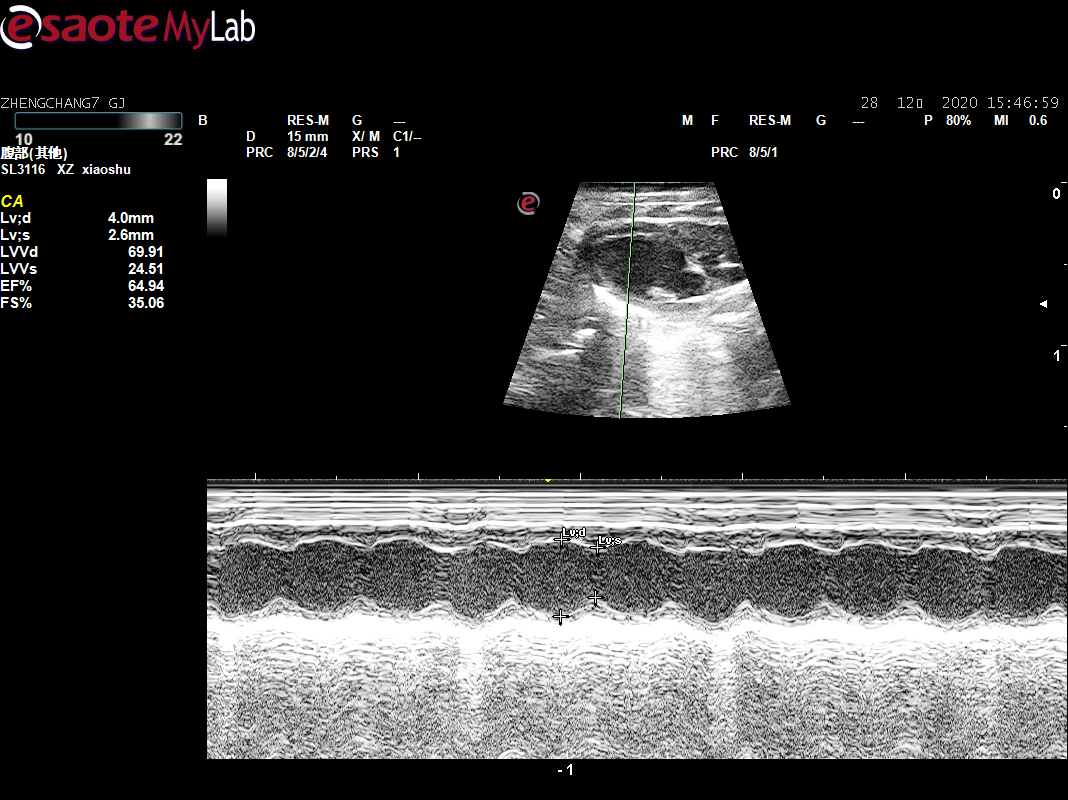


The picture above shows the FS and EF of Group S, placed after 21 days


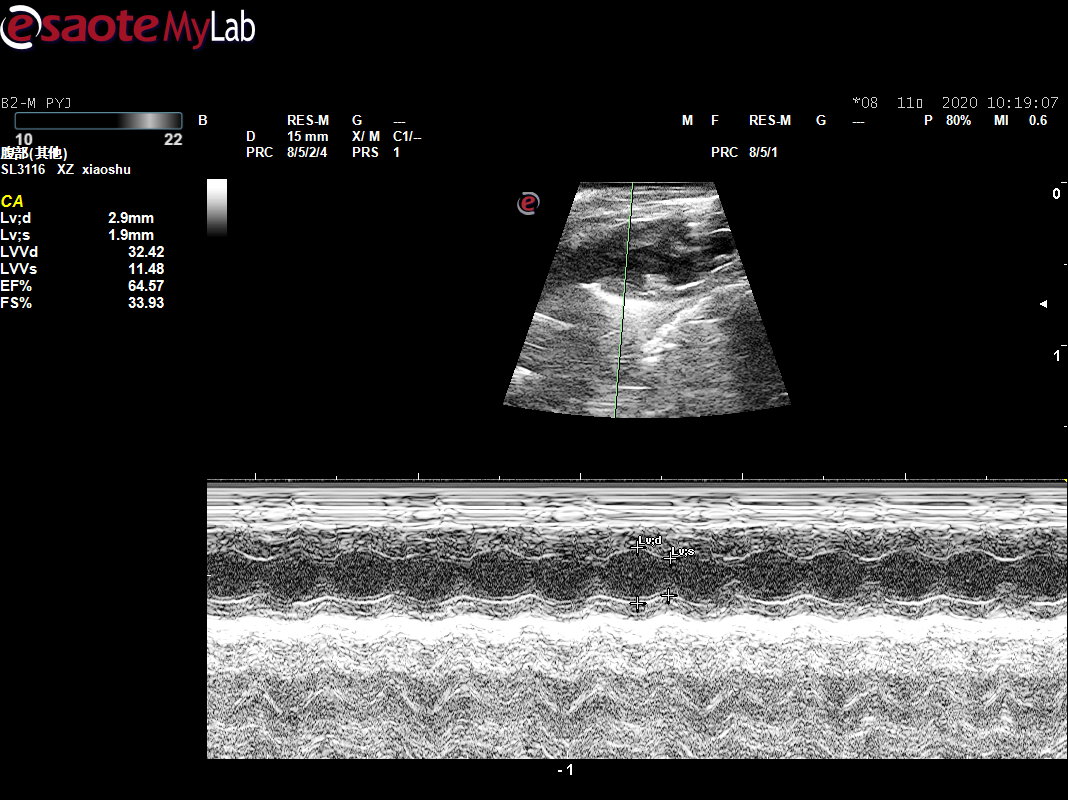


The picture above shows the FS and EF of Group A, placed after 21 days


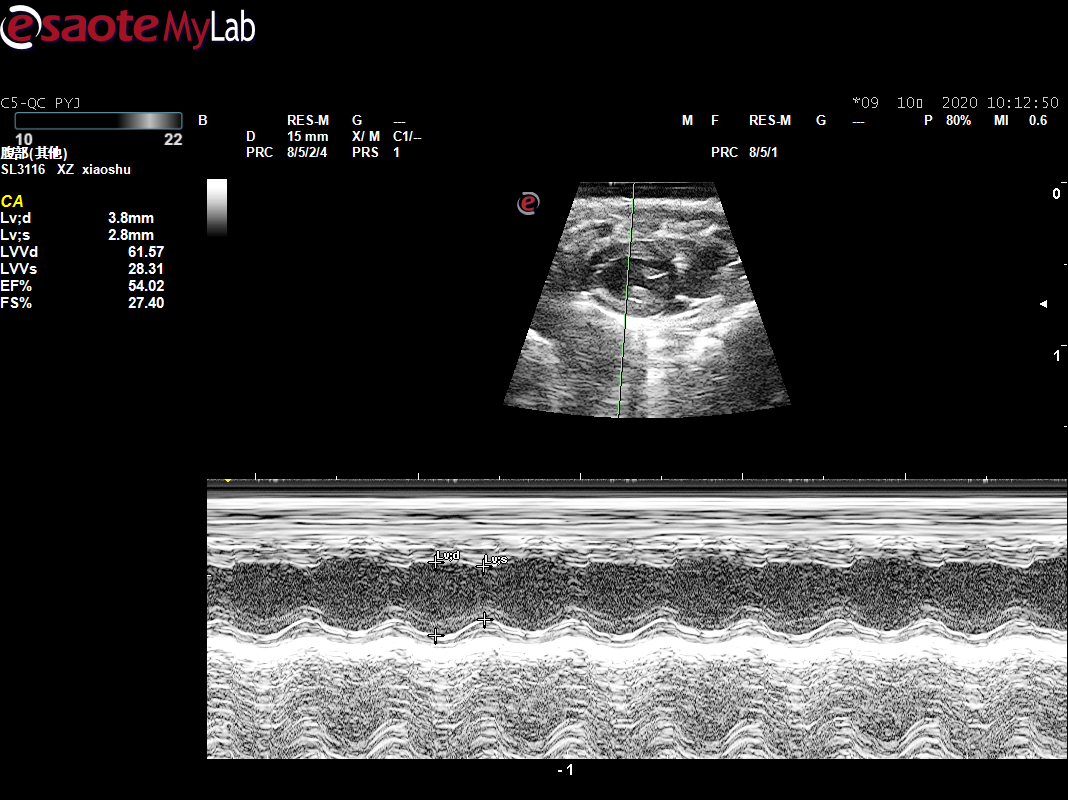


The picture above shows the FS and EF of Group B, placed after 21 days


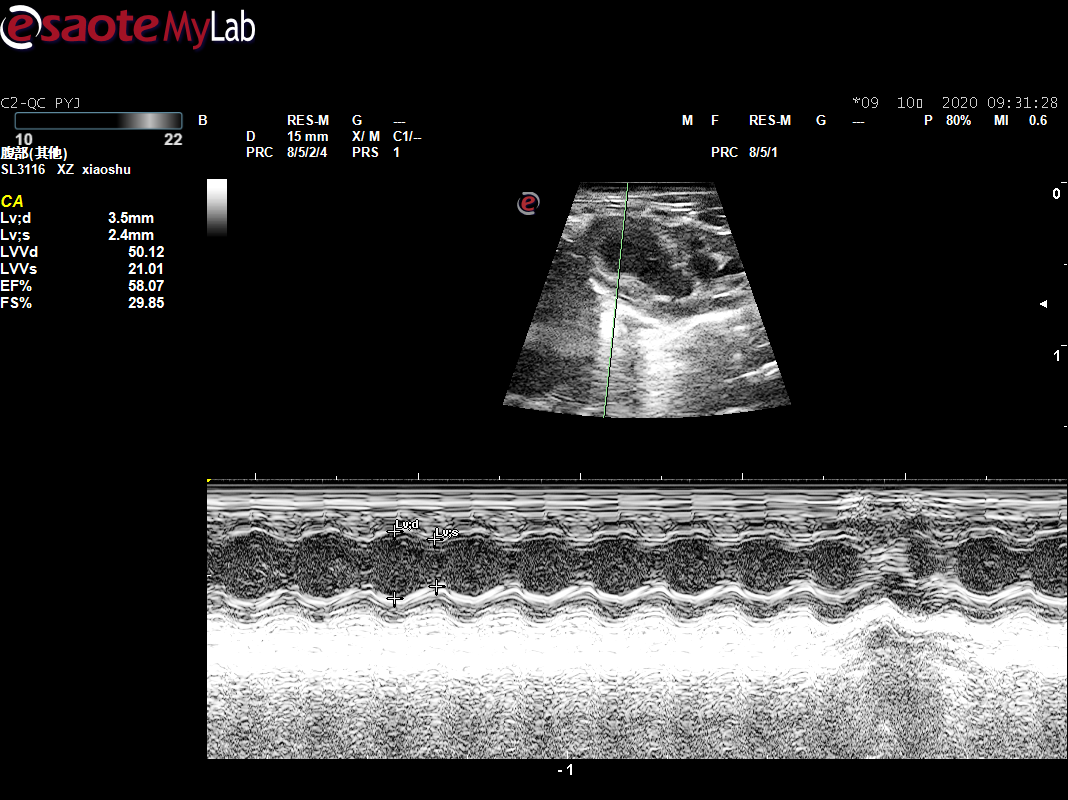


The picture above shows the FS and EF of Group C, placed after 21 days


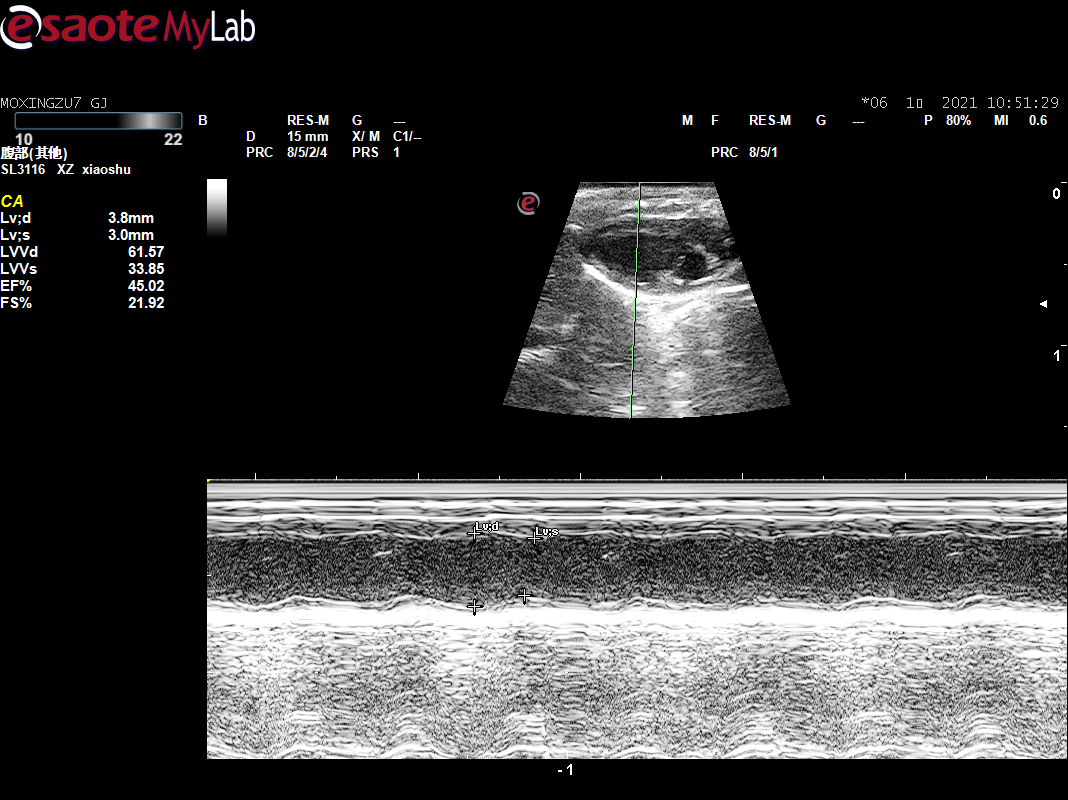


The picture above shows the FS and EF of Group D, placed after 21 days


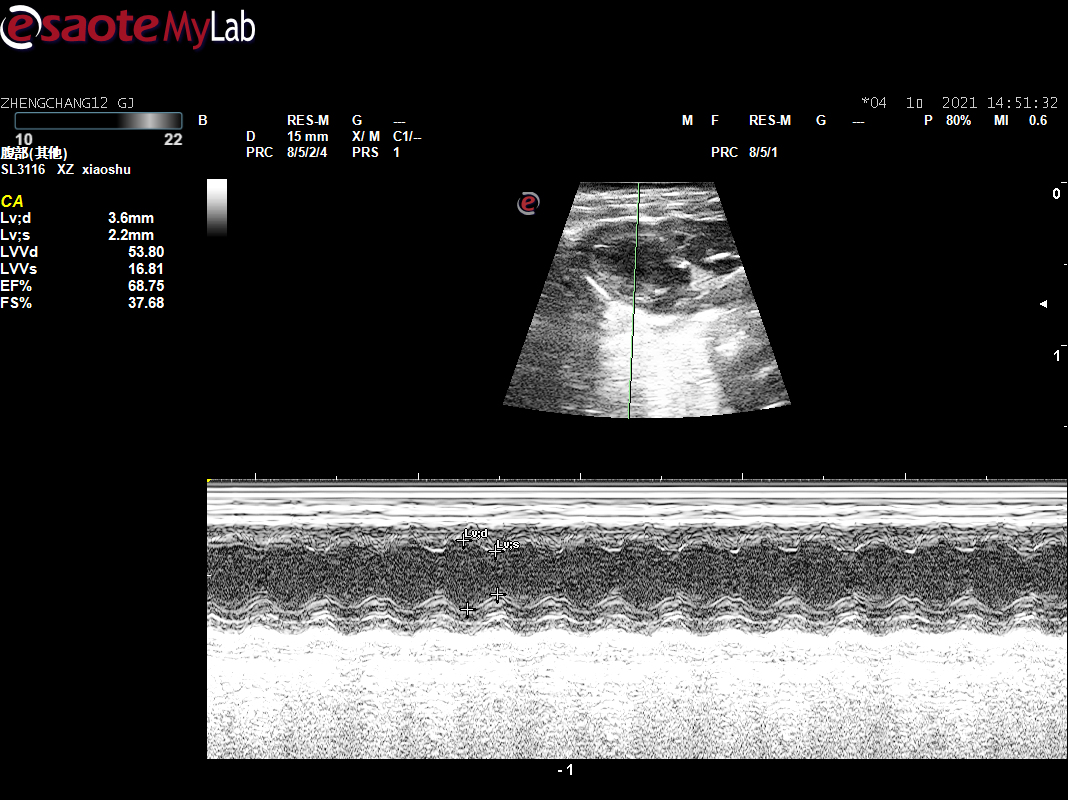


The picture above shows the FS and EF of Group E, placed after 21 days


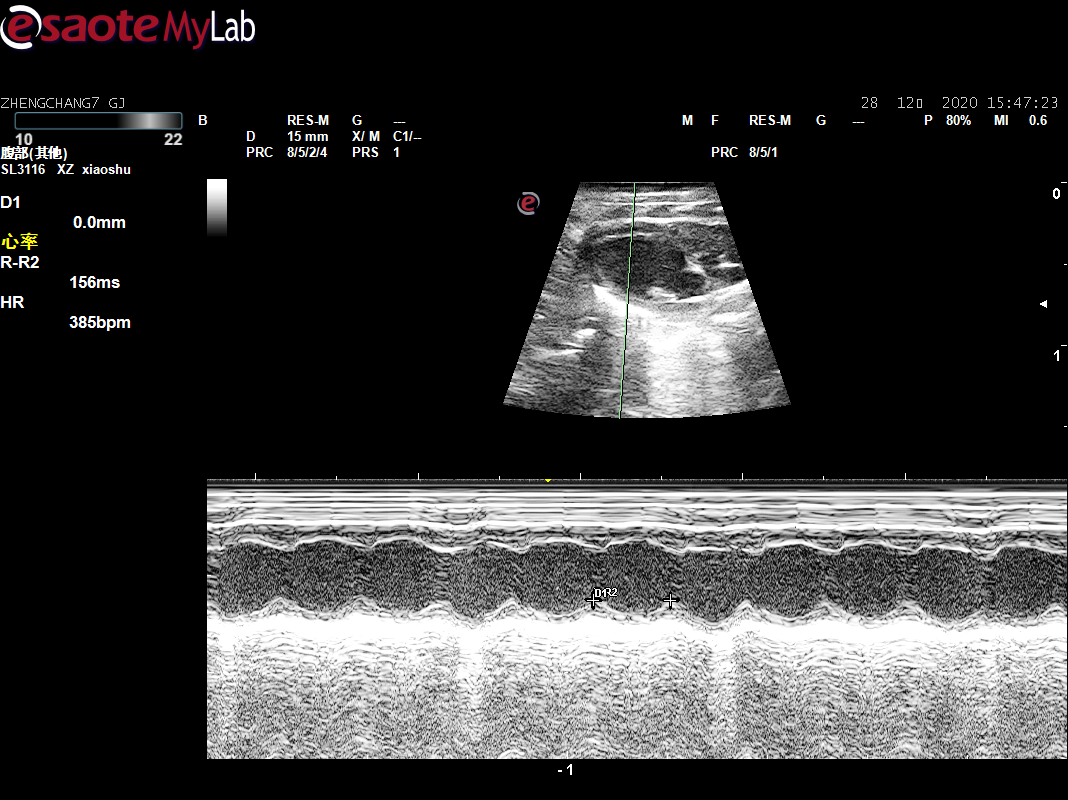


The picture above shows the HR of Group S, placed after 21 days


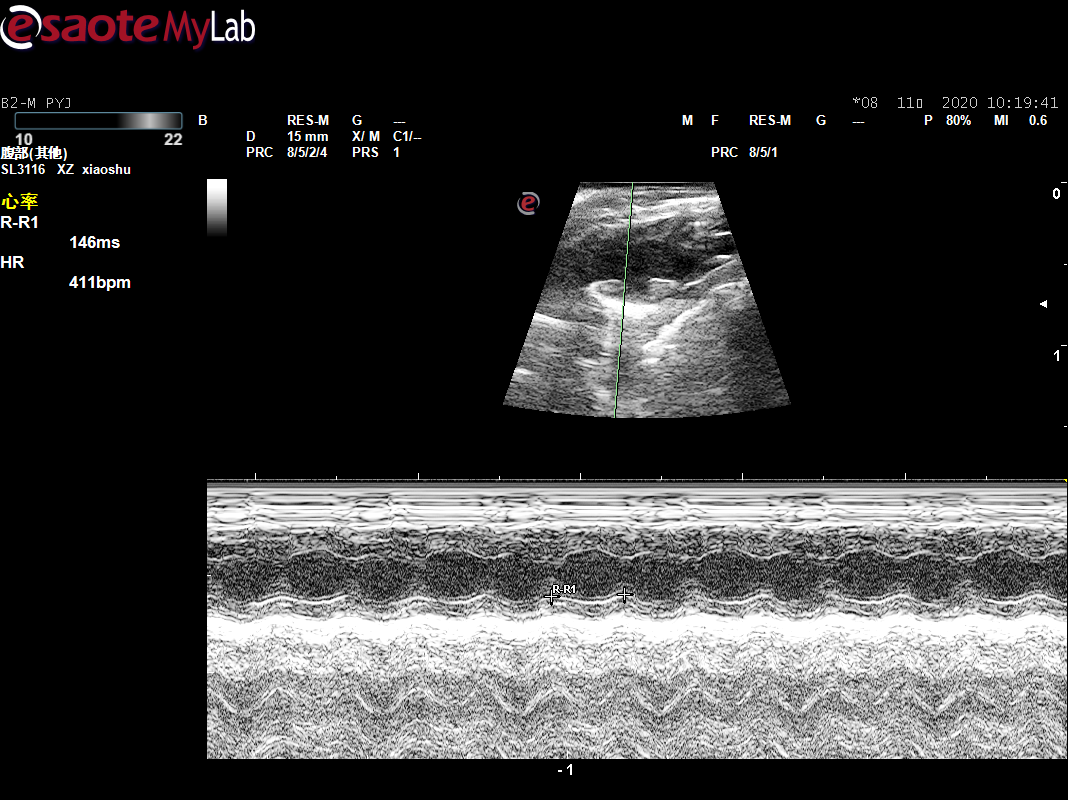


The picture above shows the HR of Group A, placed after 21 days


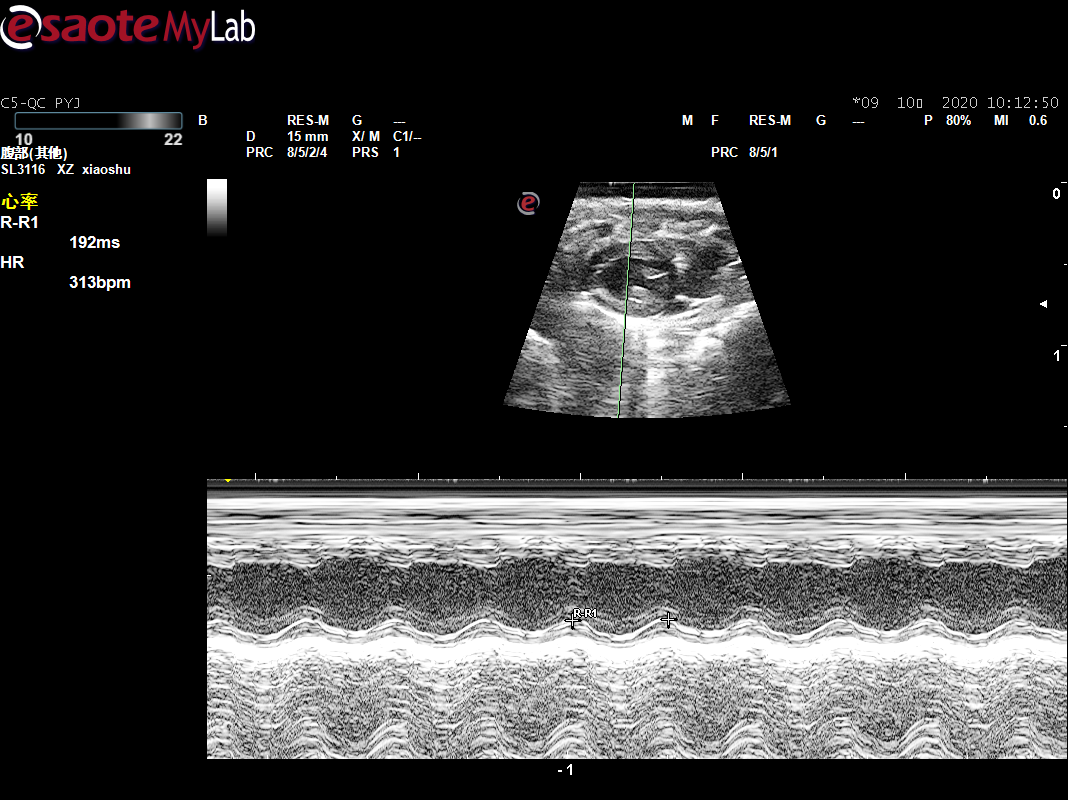


The picture above shows the HR of Group B, placed after 21 days


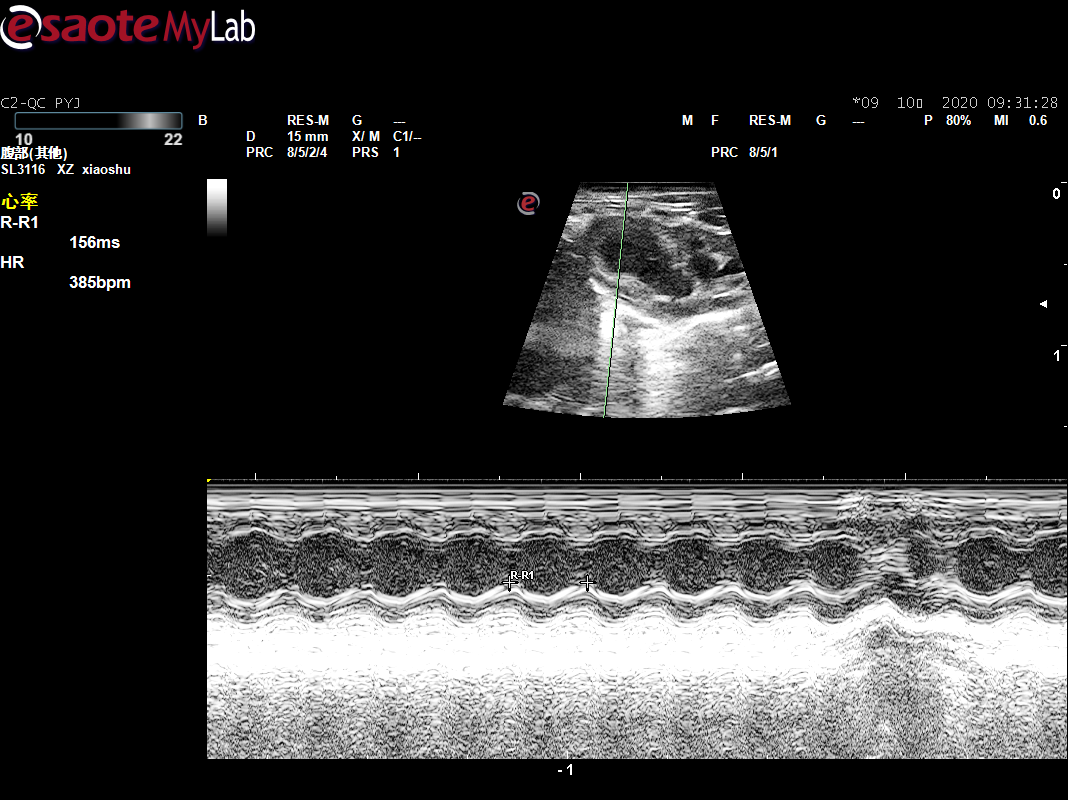


The picture above shows the HR of Group C, placed after 21 days


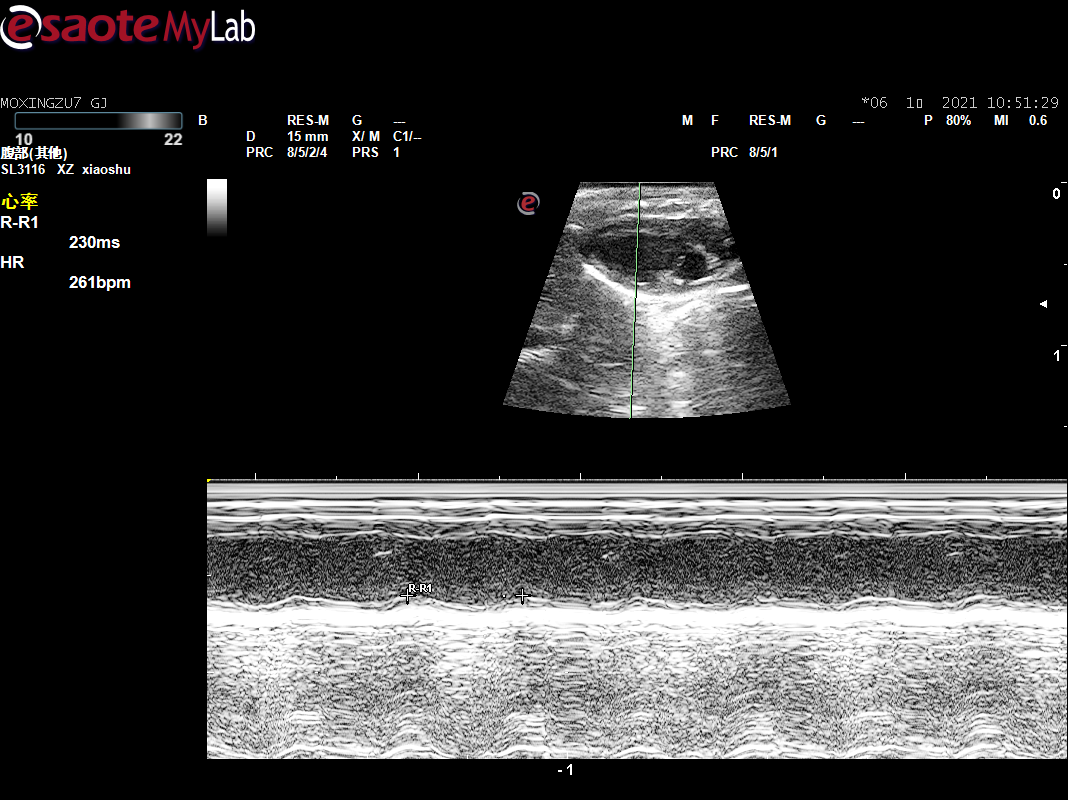


The picture above shows the HR of Group D, placed after 21 days


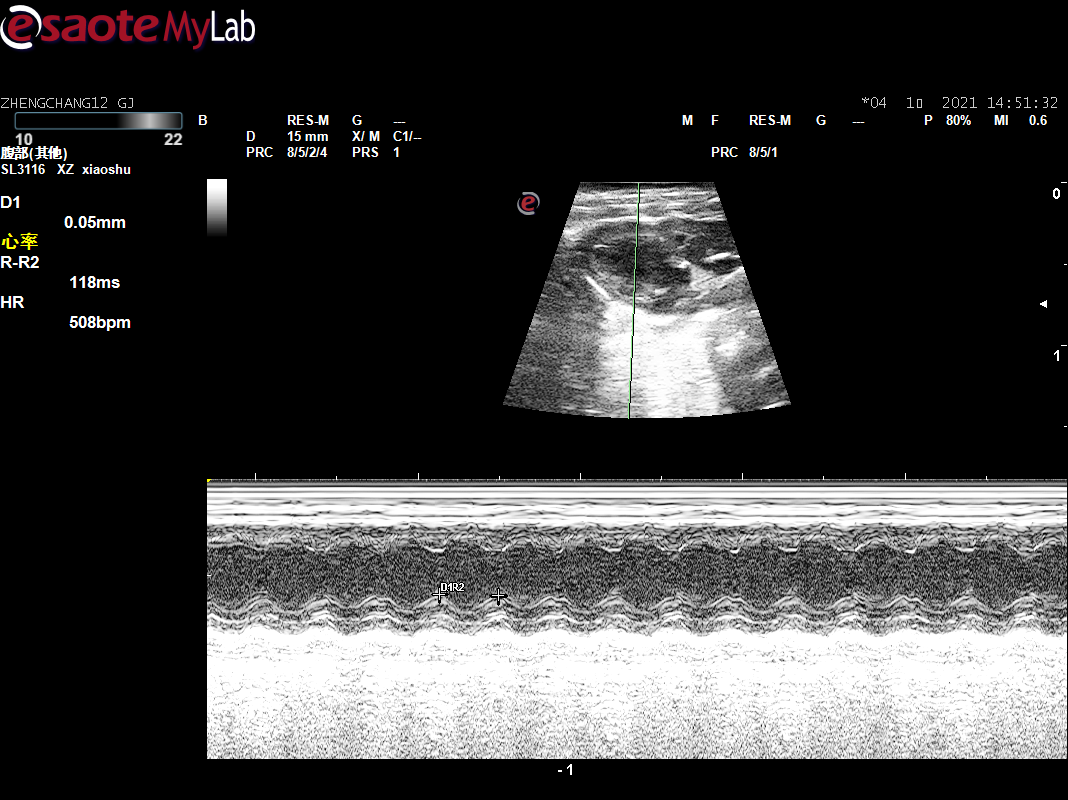


The picture above shows the HR of Group E, placed after 21 days
